# Experimental and numerical investigations on stress concentration factors of concrete filled steel tube X-joints

**DOI:** 10.1038/s41598-024-62312-8

**Published:** 2024-05-19

**Authors:** Yan Diao, Shiyi He, Yukai Wang, Liu Tu

**Affiliations:** School of Architecture and Civil Engineering, Xihua University, Chengdu, 610039 China

**Keywords:** Concrete filled steel tube, SHS-CFSHS X-joint, Stress concentration factor (SCF), Experiment, Finite element analysis, SCF formula, Civil engineering, Mechanical engineering

## Abstract

An SHS-CFSHS X-joint is fabricated by welding two square hollow section (SHS) braces to a concrete-filled square hollow section (CFSHS) chord. In this paper, the stress concentration factors (SCFs) of SHS-CFSHS X-joints are investigated through experimental tests and finite element analysis (FEA), with the hot spot stress method serving as the analytical approach. Eight specimens are designed and manufactured, with FE models built in software ANSYS. These FE models are validated against the test results. The specimens are tested under brace axial tension to determine the SCFs of the X-joints. It shows that the concrete filled in the chord effectively reduces the SCFs of the X-joints. To further explore various load conditions and the influence of the parameters, FEA is carried out and a total of 64 FE models are built. Based on the FEA results, multiple regression analysis is used to obtain the SCF formulae of SHS-CFSHS X-joints under axial tension load and in-plane bending load in the brace, respectively. Comparison and analysis of the SCF results obtained from experimental tests, the proposed formulae, and FE simulations reveal that the formulae presented in this study are both conservative and suitable for predicting SCFs.

## Introduction

Concrete-filled steel tube (CFST) members are widely used in modern structures, especially in arch and truss bridges, where the joints are composed of welded concrete-filled chord and hollow brace members^1–3^. Cyclical loading plays a dominant role in the fatigue failure of welded tubular joints. The study on the fatigue of tubular joints with hollow sections has been conducted over the past decades, and the hot spot stress method was commonly used to assess the fatigue behavior of welded tubular joints^4–7^. Hot spot method is a method to study fatigue problem, which is influenced by geometry of joints and types of loading but ignoring influence of welding process^8^. Compared with other methods like Strain-Life method and fracture mechanics approach, hot spot method has the advantages of simplicity, which provides quickly estimation of fatigue strength without consuming extensive resource. Moreover, hot spot method provides conservative and safe prediction of fatigue life, and can be well combined with other methods to predict fatigue life.

At the position of the welded joint, the distribution of hot spot stress in the weld area is relatively complex, and the degree of stress concentration can be reflected by the stress concentration factor (SCF), which plays fundamental role in hot spot method^9^. For different types of joints, such as T, Y, K, and X joints with circular or square hollow sections, the patterns of hot spot stress distribution are different; for the same type of joint, the non-dimensional parameters (i.e. β, γ and τ, where β represents the ratio of brace width to chord width, 2γ is ratio of chord width to chord thickness, and τ is brace thickness to chord thickness) and types of loading(i.e. tension, compression, and bending load) all can affect the values of SCFs^10,11^. Filling the hollow chords with concrete can significantly enhance the stiffness of the steel tube, restrict its deformation, and reduce its SCFs^12,13^. The SCFs of joints under axial loading (tension and compression respectively) and in-plane bending in the brace were compared in Ref^14^, the SCFs were larger when the brace was under axial tension than those when the brace was under axial compression or in-plane bending. Xu et al.^15^ studied the distribution pattern of hot spot stress and SCF values of T, Y, and K joints under axial tension loading, which indicates that chord thickness has a limited effect on SCFs, while joint type is the primary influencing factor. Lei Jiang^16^ used hot spot stress to investigate the SCFs in truss bridges, 1:5 scale models were built to compare three different types of K-joints, and the results revealed that using quadratic extrapolation method to calculate hot spot stress is reliable.

For the studies of SCFs in hollow X-joints, Madhup et al.^17^ studied the SCF values for cold-formed high strength steel traditional X-joints (without brace rotation or chord rotation), and member-rotated X-joints, including brace-rotated, square bird-beak and diamond bird-beak configurations. The research uncovered consistent SCF patterns across both traditional and member-rotated X-joints, revealing universal trends. In recent years, tubular joints retrofitted with fiber reinforced polymer (FRP) has also be a popular topic, Hossein Nassiraei^18,19^ investigated circular hollow steel X-joints retrofitted with FRP under out-of-plane bending and in-plane bending respectively, indicating that FRP can lower the SCFs of X-joints under both conditions, and design equations for X-joint with FRP were proposed. Through both experiment and FE analyses, Liu^20^ conducted investigations on concrete-filled rectangular hollow section X joint with perfobond leister(PBL) ribs under tension, and found that PBL ribs can enhance the ultimate strength of X-joints, as well as mitigate the stress concentration phenomenon. Jiang^21^ investigated SCFs of concrete-filled square hollow section(CFSHS) X-joints with PBL ribs under axial force and in-plane bending in both chord and brace, in which both experiments and FE models are used. It is indicated that SCFs of chord are lower than those of brace generally, because concrete can mitigate the inward buckling of side wall of the chord, and perfobond rib stiffener can further reduce outward deflection of the chord. Ran Feng and Ben Young^22^ studied the behavior of concrete-filled stainless steel tubular X-joints subjected to compression, comparing results of experiment with those calculated by design rules from CIDECT^10^, and it is demonstrated that the design rules of CIDECT are conservative for concrete-filled stainless steel tubular X-joints of square and rectangular hollow sections. The SCFs of SHS-CFSHS X-joints under axial tension in the brace were studied by the FE method in Ref^23^,and new design equations were proposed for SHS X-joints. Consequently, the investigations of SCFs of SHS-CFSHS X-joints with the brace under axial tension is notably scarce, and experiments are urgently needed.

Therefore, a study on the SCFs of SHS-CFSHS X-joints, including the experiment and FE method, is made in this paper. Eight specimens are designed to investigate the SCFs of SHS-CFSHS X-joints. Based on the experimental results, 64 FE models are established and a further study on the influence of different load conditions and non-dimensional parameters (i.e. β, γ and τ) are made. Finally, based on the results of FE analysis, multiple regression analysis is used to obtain the SCF formulae of SHS-CFSHS X-joints under axial tension and in-plane bending load.

## Experimental program

### Design of experiment

**Figure 1:**
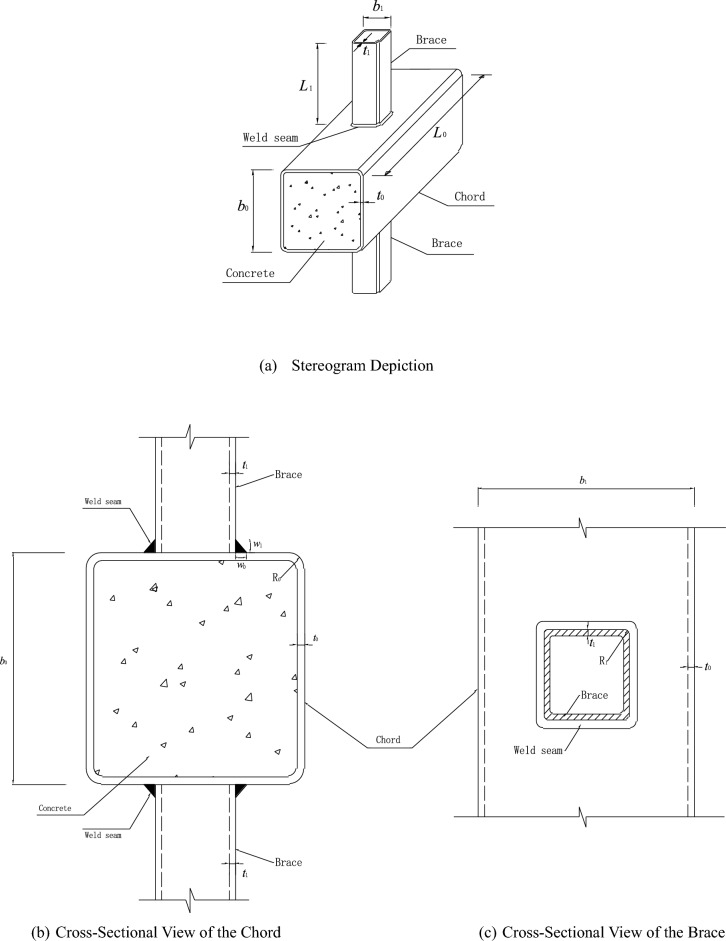
Schematic diagram of SHS-CFSHS X-joint.

In this paper, eight SHS-CFSHS X-joints are designed (Fig. 1). The detailed geometric parameters of all specimens are shown in Table 1. The ${\text{b}}_{1}$ and ${\text{b}}_{0}$ are the widths of the brace and chord, respectively; The ${\text{t}}_{1}$ and ${\text{t}}_{0}$ are the wall thicknesses of the brace and chord, respectively; The ${\text{L}}_{1}$ and ${\text{L}}_{0}$ are the lengths of brace and chord, respectively; ${\text{R}}_{1}$ and ${\text{R}}_{0}$ are the radii of cold-formed angle in the brae and chord, respectively, equivalent to 2t_0_ and 2t_1_; w_1_ and w_0_ are leg lengths of weld seam connecting the brace and chord. In addition, ${\text{w}}_{0}$ = ${\text{w}}_{1}$ = $\sqrt 2 {\text{ t}}_{1}$ conforming to the recommendation of the AWS D1.1 standard^24^ for joint penetration welds. The values of three non-dimensional parameters for SHS-CFSHS X-joints, β($= {\text{b}}_{1} /{\text{b}}_{0}$), τ($= {\text{t}}_{1} /{\text{t}}_{0}$) and 2γ($= {\text{b}}_{0} /{\text{t}}_{0}$), are listed in Table 1.

**Table 1:** Geometric parameters of specimens.

Specimens	Chord (mm)				Brace (mm)				Non-dimensional parameters		
	$b_{0}$	$t_{0}$	$L_{0}$	$R_{0}$	$b_{1}$	$t_{1}$	$L_{1}$	$R_{1}$	β = $b_{1} /b_{0}$	2γ = $b_{0} /t_{0}$	τ = $t_{1} /t_{0}$
S1	110	6	700	12	50	6	170	12	0.455	18.33	1
CS1	110	6	700	12	50	4	170	8	0.455	18.33	0.67
CS2	110	6	700	12	50	5	170	10	0.455	18.33	0.83
CS3	110	6	700	12	50	6	170	12	0.455	18.33	1
CS4	110	5	700	10	50	5	170	10	0.455	22	1
CS5	110	7	700	14	50	7	170	14	0.455	15.71	1
CS6	110	6	700	12	40	6	170	12	0.363	18.33	1
CS7	110	6	700	12	60	6	170	12	0.545	18.33	1

Specimen designations starting with the letter CS represent the X-joint specimens with the concrete filled in the chords, as the physical dimensions of the specimens vary, the names of the specimens vary from CS1 to CS7. S1 represents that there is no concrete filled in the square chord of the X-joint specimen.

To study the influence of the different parameters on the SCFs of X-joint, the specimens are well designed. CS3, CS6, and CS7 can be compared to investigate the influence of β. CS3, CS4, and CS5 can be compared to investigate the influence of 2γ. CS1, CS2, and CS3 can be compared to investigate the influence of τ. S1 and CS3 can be compared to investigate the influence of concrete filled in the chord.

According to St. Venant’s principle, the length of the chord($L_{0}$) is longer than 6 times the width of the chord($b_{0}$), the length of the brace($L_{1}$) is longer than 3 times the width of the brace($b_{1}$), so that the joint area is far enough from the supports to avoid the influence of the end constraints on the stress^8^. Hence, the $L_{0}$ is 700 mm and $L_{1}$ is 170 mm for all specimens in this paper.

### Material properties

20# steel is used to establish steel tubes in this research, which has 0.2% of carbon. Steel tubes in this research follow the standard of Cold Forming hollow sectional steel for general structure(GB/T 6728)^25^, According to the requirements of the Standard for Test Methods of Mechanical Properties on Ordinary Concrete (GB/T50081)^26^, the 150 mm $$\times$$ 150 mm $$\times$$ 150 mm concrete cubes have the average compressive strength ($$f_{cu}$$) of 54 MPa, and the prismatic concrete 150 mm $$\times$$ 150 mm $$\times$$ 300 mm has the average modulus of elasticity of $$3.35 \times 10^{4}$$ MPa, which were made in the same condition and cured for 28 days. According to the requirements of Metallic Materials-Tensile Testing-Method of Test at Ambient Temperature (GB/T228)^27^, the measured yield strength (*f*_*y*_) and ultimate strength (*f*_*u*_) of the steel tube were 280 MPa and 440 MPa, respectively. The elastic modulus ($$E_{s} )$$ of steel tube is $$2.06 \times 10^{5}$$ MPa and Poisson’s ratio is 0.283. Previous research has shown that the concrete grade has little effect on the SCFs of concrete-filled steel tube joints^28,29^.

### Specimens preparation and test procedure

At first, pre-test of concrete was conducted to ensure the concrete attains the required strength, and the concrete is cured in natural condition for 28 days, measured as an ideal strength of 54 MPa. In addition, to make sure the concrete is combined with steel tube well, the concrete has relatively high slump value and fluidity. After that, this kind of concrete was used for establishing concrete-filled steel tubes in this paper.

Hollow steel tubes were made by local factory. The chord and brace were welded together by gas metal arc welding method, and full penetration weld was used, with a leg length of $\sqrt 2$ times wall thickness of brace, respectively, following the Chinese Standard-Welding Code for Steel Structures (GB50661-2011)^30^. Two plates, with thickness of 20 mm, and two steel bars, with diameter of 20 mm, were welded to both ends of the brace tubes to enable clamping by the test machine's clamps. Prior to bonding the strain gauges, the surfaces of the steel tubes were meticulously cleaned using abrasive paper and 75% alcohol to ensure effective adhesion.

**Figure 2:**
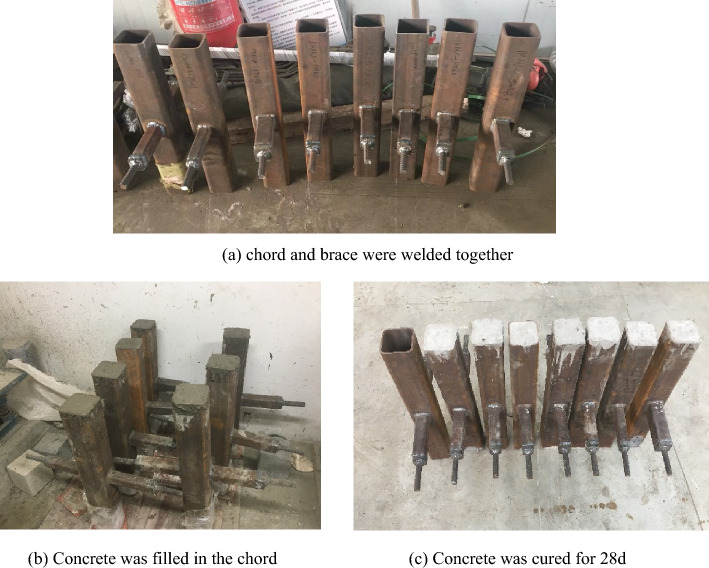
Fabrication process of Specimens.

After the fresh concrete and steel tubes were prepared, appropriate formwork and supports were set up to securely hold the steel tubes upright during the concrete pouring process, ensuring their stability and preventing any movement. Subsequently, the concrete was poured gradually and slowly into the steel tubes, with the aim of minimizing the risk of voids and air pockets. Vibrational techniques were employed to compact the concrete effectively within the steel tubes, guaranteeing optimal density and a strong bond with the steel tube. Following these meticulously executed procedures, the concrete was filled within the chord and allowed to naturally cure at a temperature of 20 °C for a period of 28 days, during the curing process, a hygrometer was employed to monitor the room's humidity levels, while the specimens required watering every 24 h to prevent concrete cracking. After 28 days, the formation of specimens of SHS-CFSHS X-joints was accomplished as shown in Fig. 2.

**Figure 3:**
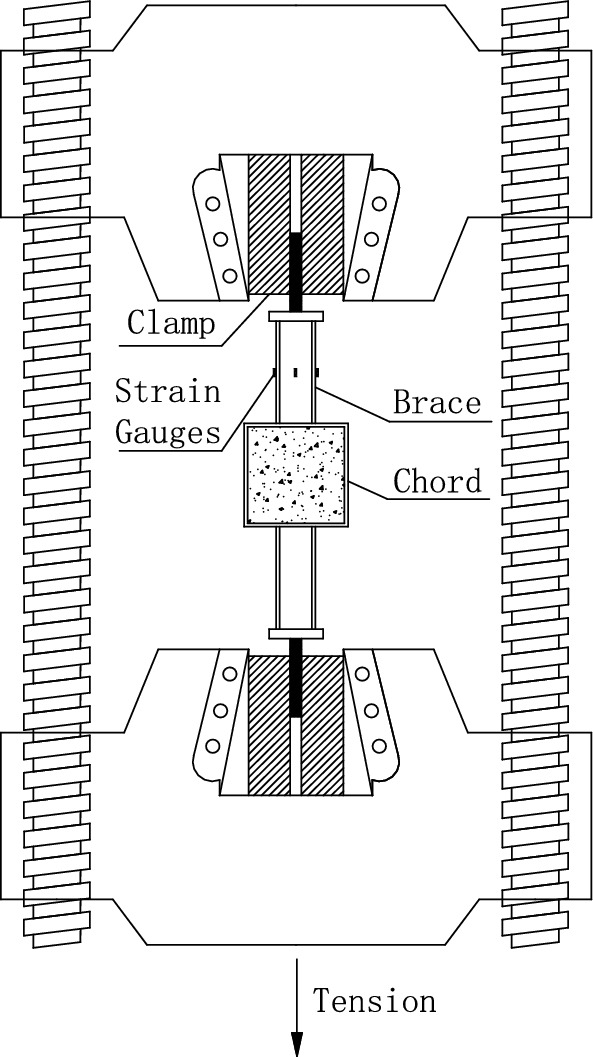
Illustrative drawing of loading.

**Figure 4:**
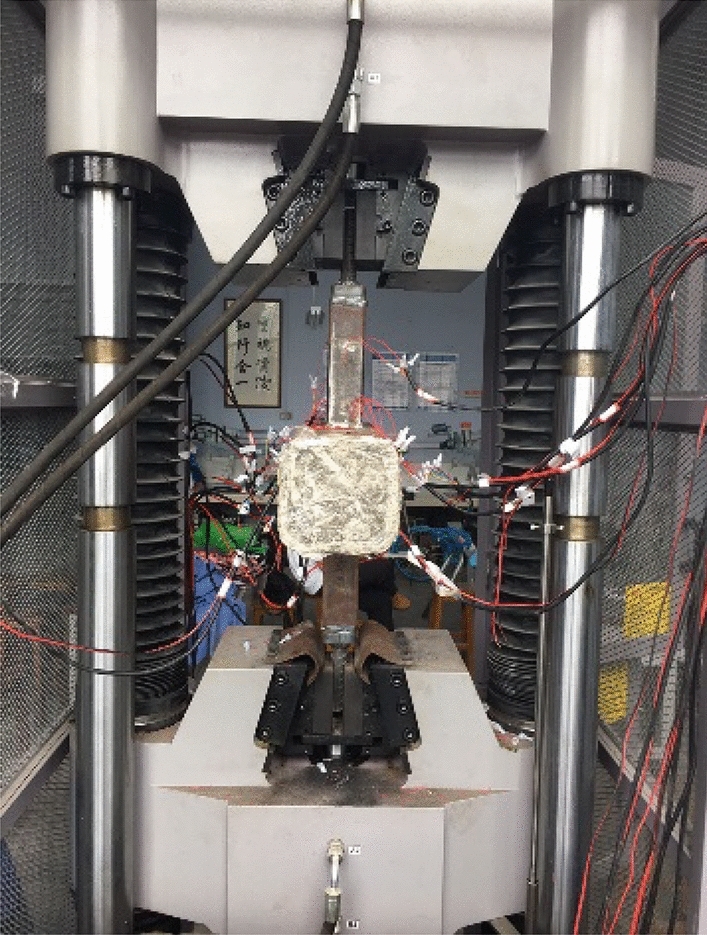
Test setup for hot spot stress measurements.

In the test, axial tensions were applied to the brace members. The tensions were limited to a certain range that kept the joints in the elastic stage. The progressive loading method was used in the test, following the steps of 0 → 20kN → 40kN, with a loading speed of 50N/s. When loading reached 40kN, the loading was kept unchanged for 2 min, so that the data could be recorded conveniently. The illustrative drawing of loading is shown in Fig. 3 and the test process is shown in Fig. 4.

**Figure 5:**
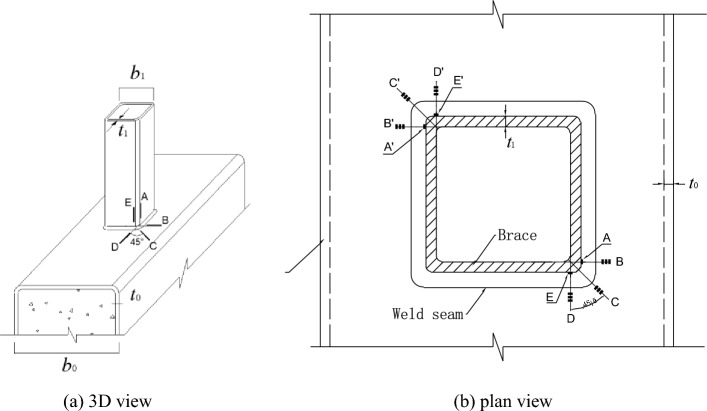
Extrapolation lines and strain gauges arrangement.

Researchers have studied the locations of hot spot stress on tubular joints, for CHS joints, hot spot stress locations not only appear at the crowns and saddles of chord and brace, but also locations of welded intersecting lines^31^. For CHS joints, according to CIDECT Design Guide No.8^25^, ten extrapolation lines were arranged, which were lines $A$, $A^{\prime}$, $E$, $E^{\prime}$ in the brace member and lines $B$, $B^{\prime}$, $C$, $C^{\prime}$, $D$, $D^{\prime}$ in the chord member (Fig. 5). When the chord is filled with concrete, the same locations have been proposed to test the hot spot strains and calculate the SCFs^28^.

For SHS joints, the quadratic extrapolation method is commonly used to determine the hot spot strain because of the strong non-linear strain distribution along the line perpendicular to the weld toe^2^, so quadratic extrapolation was used in this paper to calculate hot spot strain of SHS-CFSHS X-joint.

At each extrapolation line, three strain gauges were placed perpendicular to the weld toe to measure the strains of specimens. According to Ref.^10^, the distance ($$L_{min} )$$ of the first strain gauge from the weld toe was $$0.4t_{0}$$(for chord) or $$0.4t_{1}$$(for brace), and the minimum value for $$L_{min}$$ was 4 mm for excluding the notch effect of the weld. The maximum distance ($$L_{max} )$$ of the last gauge from the weld toe was $$L_{min} + t_{0}$$ (for chord) or $$L_{min} + t_{1}$$ (for brace). In addition, four strain gauges were placed in the middle of the brace away from the end of the brace and brace-chord intersection to test the nominal strains (Fig. 3). The arrangement of extrapolation lines and strain gauges for hot spot strain measurements was shown in Fig. 5.

## Test results and discussion

**Figure 6:**
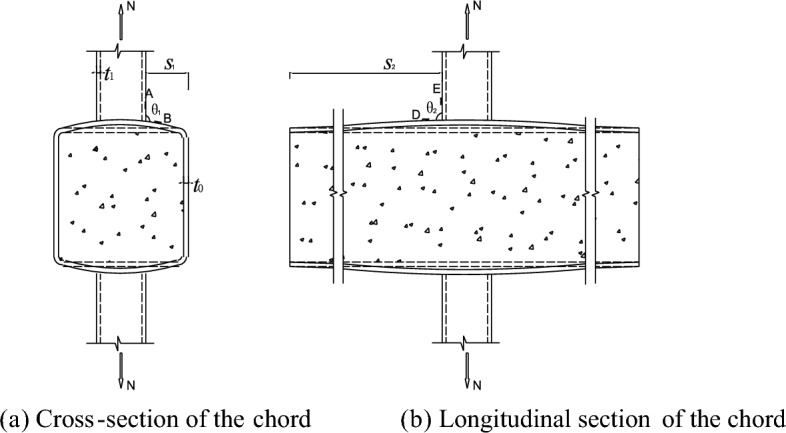
The possible elastic deformation of structure in different views.

Figure 6 shows the possible elastic deformations of the joints, when the braces are subjected to the axial tension, the braces will drive the top and bottom walls of the chord deformation, the deformations of cross-section and longitudinal-section of the chord are shown in Fig. 6a,b, respectively.

The $\theta_{1}$ and $\theta_{2}$ are the angles between the brace wall and the chord wall in cross-section and longitudinal-section of the chord, respectively. If the brace stretches and the chord deforms, $\theta_{1}$ is greater than $\theta_{2}$, it means the change in angle of the cross-section of the joint is greater than the change in angle of the longitudinal-section.

**Figure 7:**
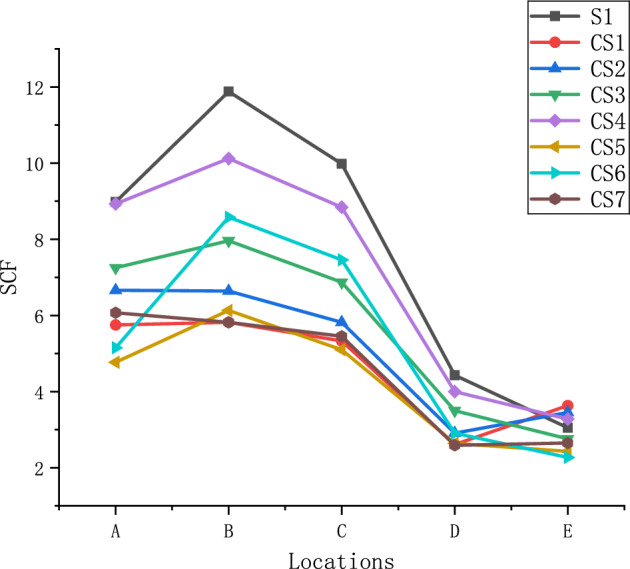
SCFs of specimens.

**Figure 8:**
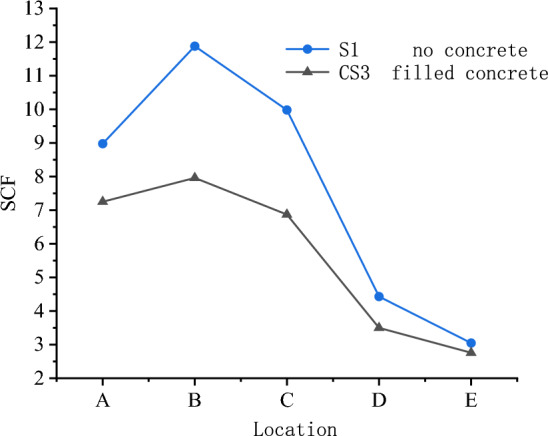
Comparison of specimens with and without concrete.

**Figure 9:**
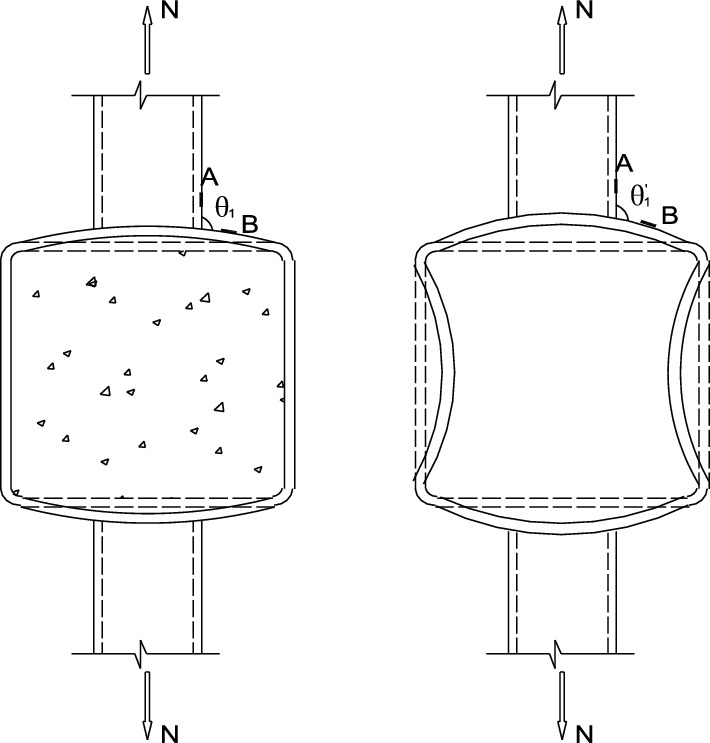
The comparison of possible deformation of joints with and without filled concrete.

**Figure 10:**
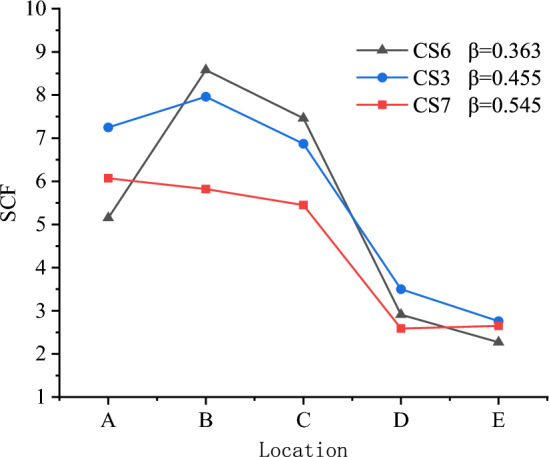
Effect of β on SCFs.

**Figure 11:**
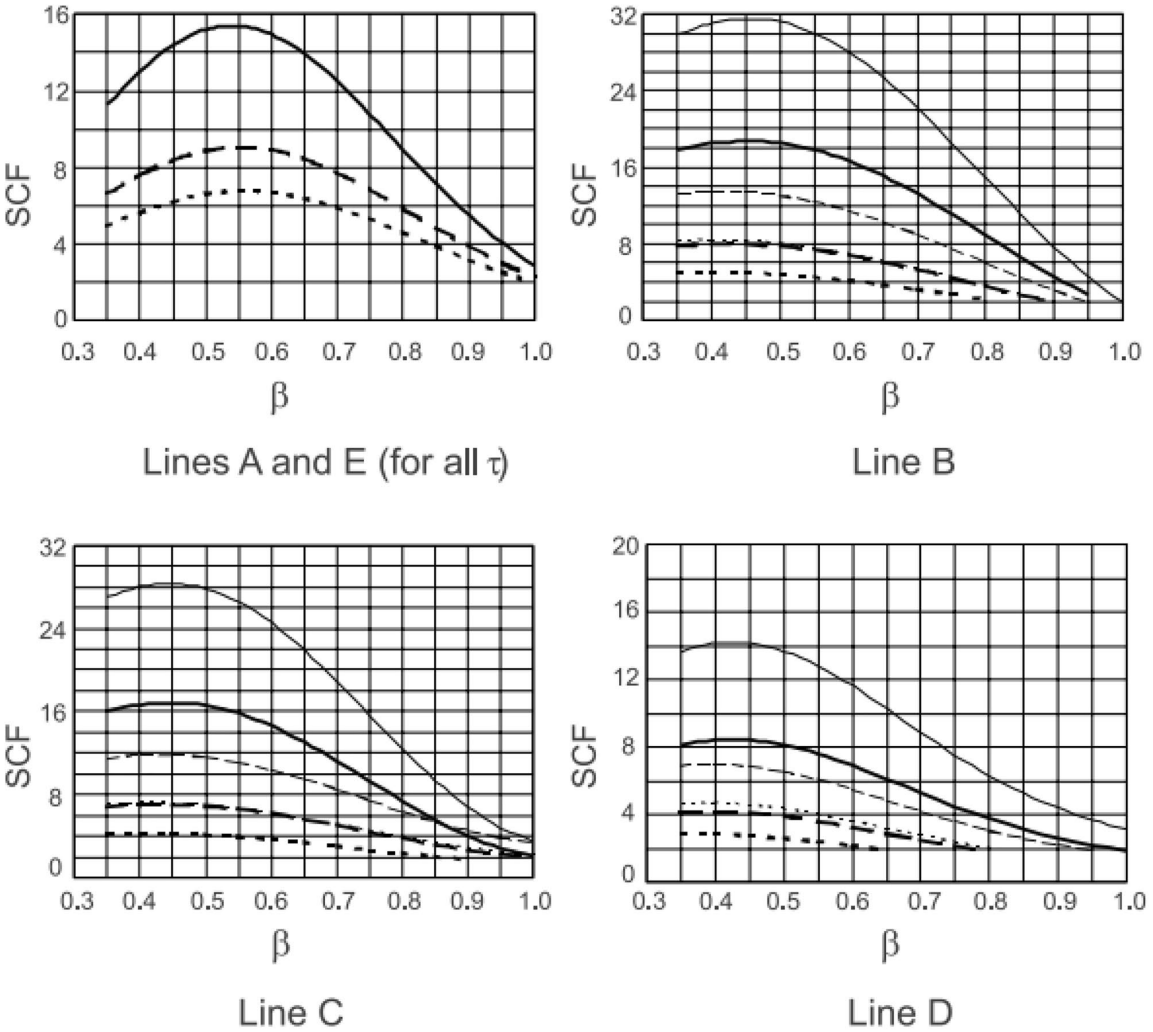
SCFs for X-joints of square hollow section loaded by an axial force on the brace^10^.

**Figure 12:**
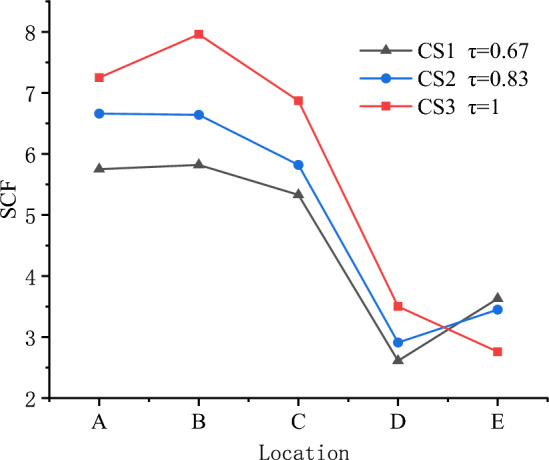
Effect of τ on SCFs.

**Figure 13:**
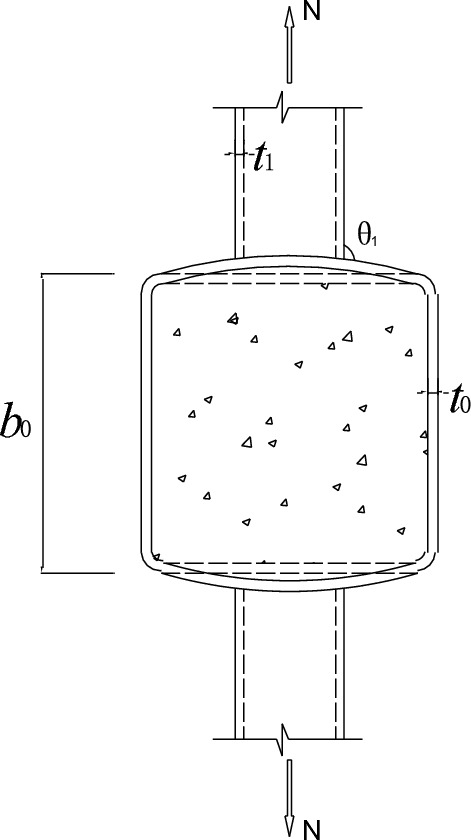
The possible deformation of cross-section of the chord.

**Figure 14:**
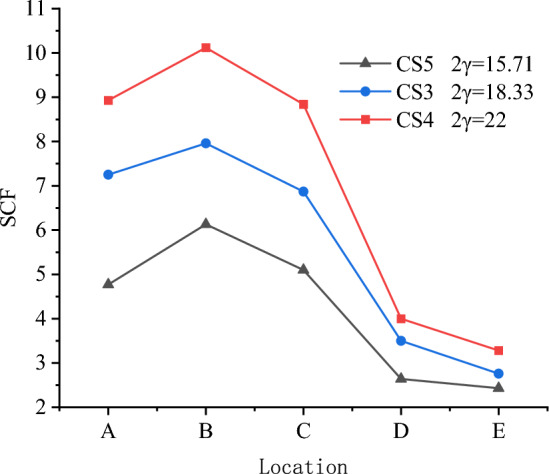
Effect of 2γ on SCFs.

$S_{1}$ is the distance from the brace to the side wall of the chord, the side walls of the chord can limit the displacement and deformation of the top plate of the chord, so the side walls are the constraints of the top plate. $S_{2}$ is the distance from the brace to the end of the chord. As the brace along the axial stretching, the shape and angle of the cross-section of the joint varies more than that of the longitudinal-section, but is more restricted because $S_{1}$ is much smaller than $S_{2}$. So the points in the cross-section of the structure have higher stress level than the ones in the longitudinal-section. The stress of points in line A and line B must be higher than the stress of points in line D and line E (Figs. 7, 8, 9, 10, 11, 12, 13, 14). The location of the points in line C are between the line A, B and line D, E, so the magnitude of the stress of the points in line C also lies between the two. The stress distributions of all the specimens in the test were consistent with the analysis, as shown in Fig. 8, Fig. 10, Fig. 12 and Fig. 14, as well as those of Ref^23^. Notably, similar stress distributions occur in high-strength steel and stainless steel tubular X-joints without concrete^32,33^, indicating a universal pattern in the stress distribution of X-joints.

Strain concentration factor (SNCF) is the ratio of hot spot strain to nominal strain, and the strains can be tested and obtained through the experiment, so the SNCF can be determined first. Then the SNCF was converted to the stress concentration (SCF) by Eq. 1^34^:

1 $${\text{SCF}} = { 1}.{\text{1SNCF}}$$

The original strains were measured by strain gauges under 40kN tension. By using quadratic extrapolation method, average nominal and hot spot strains are illustrated in Table 2. Figure 7 is the result of SCFs for the lines $A$, $B$, $C$, $D$ and $E$ for all specimens, which are actually the average values of SCFs at lines $A\left( {A{^{\prime}}} \right)$, $B\left( {B{^{\prime}}} \right)$, $C\left( {C{^{\prime}}} \right)$, $D\left( {D{^{\prime}}} \right)$ and $E\left( {E{^{\prime}}} \right)$, respectively, because the arrangements of lines $A$, $B$, $C$, $D$, $E$ and lines $A{^{\prime}}$, $B{^{\prime}}$, $C{^{\prime}}$, $D{^{\prime}}$, $E{^{\prime}}$ are exactly symmetrical. To study the influence of the different parameters on the SCF of X-joint, the curves of specimens are divided into several groups for comparison, which are shown in Fig. 8, Fig. 10, Fig. 12, and Fig. 14.

**Table 2:** Hot spot strains using quadratic extrapolation method.

Specimen number	Line A	Line B	Line C	Line D	Line E	Nominal strain
S1	1632.97	2160.32	1814.82	805.58	554.63	200.03
CS1	1414.29	1431.51	1310.99	641.97	892.85	270.56
CS2	1226.05	1222.36	1071.41	535.70	635.11	202.5
CS3	1223.08	1342.85	1158.97	590.45	465.61	185.57
CS4	1668.53	1890.88	1651.71	747.38	612.85	205.53
CS5	705.74	906.96	754.57	390.60	359.53	162.75
CS6	1089.69	1815.45	1578.47	615.73	480.31	232.75
CS7	703.95	674.96	632.05	300.37	307.33	127.57

Figure 8 shows the effect of concrete on the SCFs of the X-joint. The sizes of braces and chords of S1 and CS3 are completely the same, the difference is that CS3 is filled with concrete and S1 was not filled. The distribution patterns of SCFs for the two specimens were similar. The maximum SCF occurs in the chord at line B for S1 and CS3, and the values of SCFs at lines $A$, $B,$ and $C$ are significantly higher than the values of SCFs at lines $D$ and $E$. Compared with S1, the value of SCF at each line of CS3 is smaller than that of S1. It shows that the filled concrete in the chord, as the support of the chord wall, enhances the stiffness of the chord and effectively reduces the values of SCFs at X-joint. The maximum value of SCF of S1 is 11.88 and the one of CS3 is 7.96, which decreases by 33%.

As shown in Fig. 9, the joint without filled concrete have greater deformations of the chord, and the change in shape and angle lead to greater stress concentrations. Notably, the scale of Figs. 9 and 13 is amplified in order to clearly illustrate the deformations.

Figure 10 shows the effect of β on the SCFs of the X-joint. For CS6, CS3, and CS7, the distribution patterns of SCFs are similar, the maximum SCFs occur at line B in the chord for these three specimens, and the values of SCFs at lines $A$, $B,$ and $C$ are significantly higher than the values of SCFs at lines $D$ and $E$.

The SCF values of lines B, C, and D, which are in the chord, decrease as β increase from 0.363 to 0.545The maximum value of SCF decreases from 8.58 to 5.82 for CS6, CS3, and CS7. Lines ${\text{A}}$, ${\text{E}}$ are in the brace, it can be seen that the highest SCFs in the brace are found for medium β ratio (0.455).

According to the Ref.^10^, for rectangular hollow section X-joints, the highest SCFs in the brace are found for medium β ratio (β is from 0.35 to 1.0, when β is about 0.55, the SCFs of line A, E are the highest). In this test, the chords are filled with concrete, the SCFs of all points decrease, but the stress distribution pattern is similar with the X-joints without concrete. The β of CS6, CS3, and CS7 are 0.363, 0.455, and 0.545, respectively, the SCFs of line A, E of CS6 are minimum because its value of β is far from the middle value. The highest SCFs of line A, E occur when β = 0.455, affected by concrete, which makes the highest SCFs transfer from β = 0.55 to β = 0.455.

In Fig. 11^10^, for lines B, C, and D, the highest SCFs occur when β = 0.45, compared with the highest SCFs of lines A, E, the β decreases. So, in our test, the SCFs of lines B, C, and D are the highest when β = 0.363, which decreases from 0.455 to 0.363, the pattern is the same with Ref.^10^.

Figure 12 shows the effect of τ on the SCFs of X-joint. The maximum values of SCFs are 7.96, 6.66, and 5.82 for CS1, CS2, and CS3. The maximum SCFs occur at line B in the chord for CS1 and CS3, and occurr at line A in the brace for CS2. The values of SCFs at lines ${\text{A}}$, ${\text{B}}$, and ${\text{C}}$ are significantly higher than the values of SCFs at lines ${\text{D}}$ and ${\text{E}}$ for these three specimens.

The values of SCFs in Lines ${\text{A}},{\text{ B}}$, ${\text{C and D}}$ increase as the τ increase from 0.67 to 1, but the values of SCFs in Lines ${\text{E}}$ decreased. The locations of maximum SCF do not change as the τ change, the maximum SCF in the brace always occur at line A, and the maximum SCF in the chord always occur at line B.

Figure 13 shows the possible deformation of cross-section of the chord. When τ(τ = $t_{1} /t_{0}$) increases, it means the brace becomes stronger and chord becomes weaker. When the braces are subjected to axial tension and stretching, the chord is more prone to deformation. The same principle applies to 2γ = $b_{0} /t_{0}$(width-thickness ratio of chord). When 2γ increases, if $b_{0}$ remains unchanged, the thickness of the chord $t_{0}$ decreases. This means the chord becomes weaker compared to the brace, and when the braces are subjected to axial tension and stretching, the chord is more prone to deformation. Therefore, the SCFs of lines A, B, C, D, and E increase as the τ or 2γ increases.

Figure 14 shows the effect of 2γ on the SCFs of the X-joint. The maximum values of SCFs are 10.12, 7.96, and 6.13 for CS4, CS3, and CS5. The maximum SCF occurred at line B in the chord for all three specimens. The values of SCFs at lines $A$, $B$, $C$, $D$, and $E$ increase as 2γ increase from 15.71 to 22, and the locations of maximum SCF do not change as the 2γ change, the maximum SCF in the brace always occur at line A, and the maximum SCF in the chord always occur at line B.

## Finite element analysis

The experiment in this paper has studied the SCFs with or without concrete, as well as the effects of β, γ, and τ on SCFs. To make a further study on the SHS-CFSHS X-joints with different parameters, and obtain a universal rule of SCFs, ANSYS workbench was used to establish FE models with different parameters to investigate the SCFs of X-joints under axial tension loading.

### FE models

**Figure 15:**
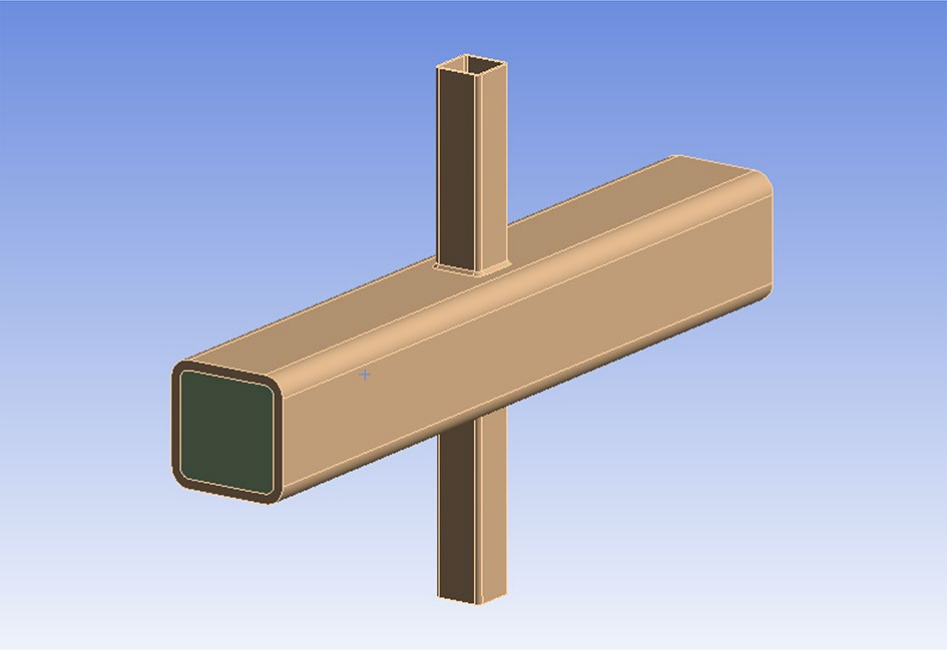
FE model of SHS-CFSHS X-joint.

The measured material properties in the test were adopted for the FE models. The yield strength (*f*_*y*_) and ultimate strength (*f*_*u*_) of steel tubes were 280 MPa and 440 MPa, respectively. The steel in the FE models exhibits a Young’s modulus of $2.06 \times 10^{5}$ MPa, bulk modulus of 1.58 $\times$ 10^4^ MPa, and shear modulus of 7.99 $\times$ 10^4^ MPa. The core concrete filled in the chord is C50, with a Young’s modulus of $3.35 \times 10^{4}$ MPa, bulk modulus of 1.72 $\times$ 10^4^ MPa, and shear modulus of 1.5 $\times$ 10^4^ MPa. The Poisson's ratios of steel and concrete are taken as 0.3 and 0.2^36^, respectively. The FE model of the SHS-CFSHS X-joint is shown in Fig. 15.

**Figure 16:**
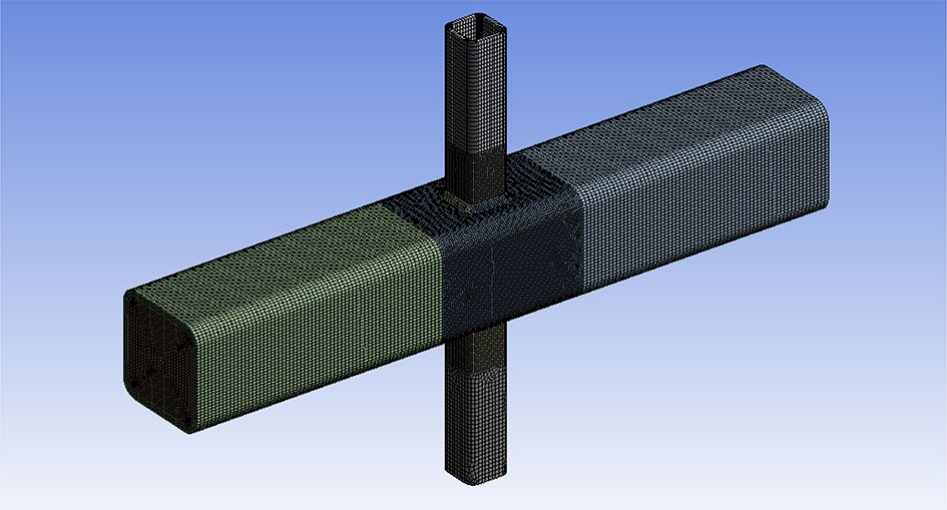
FE Mesh of X-joint.

In this study, the choice of element types, mesh sizes, and convergence criteria in the FE analyses is carefully justified to balance computational efficiency with accuracy. Since the primary focus is on investigating SCFs near the weld at the intersection of the brace and chord, a strategic approach is adopted to optimize the FE model. To achieve this, the model is divided into two distinct zones for meshing. The first zone, located away from the intersection of the brace and chord, utilized a mesh size of 4 mm. This larger mesh size helped minimize computational costs while still capturing the overall behavior of the structure accurately. The second zone encompasses the critical intersection area, where high stress concentration phenomena are usually anticipated. Here, a finer mesh size of 2 mm is employed to ensure sufficient resolution and accuracy in capturing the localized effects of stress concentration. The choice of element type is crucial in accurately representing the complex behavior of the concrete-filled steel tubes. Solid186, characterized by 20 nodes and supporting capabilities of plasticity, elasticity, large deflection, and large strain, are selected to simulate the core concrete, steel tube, and weld. The use of Solid186 elements allowed for a realistic representation of material behavior and structural response. Furthermore, a quadratic element order was chosen over a linear element order to enhance the accuracy of the FE models, particularly in modeling curved boundaries and nonlinear behavior. The mesh configuration of the FE models is illustrated in Fig. 16.

**Figure 17:**
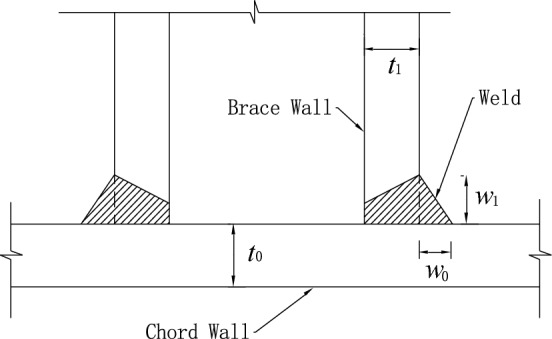
The weld sizes.

**Figure 18:**
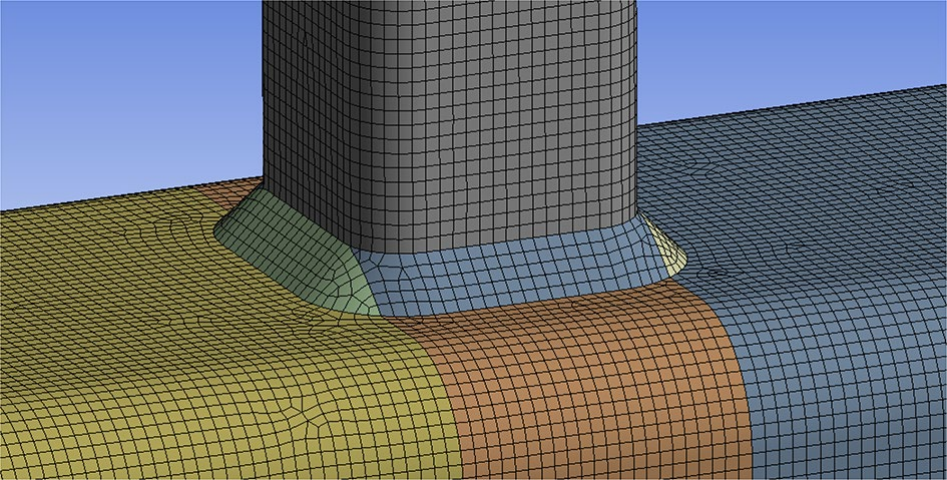
The profile of weld in FE models.

Previous studies indicated that weld profile at the intersection of brace and chord significantly influences the SCFs of the joint^35^. In a related study, M.M.K Lee built different sizes of welds in FE models to investigate the effect of weld shape on the SCFs of the joint^36^. In FE models, the radii of cold-formed angles of brace and chord were set as *R*_*1*_ and *R*_*0*_, respectively, *R*_*1*_ = *2t*_*1*_, *R*_*0*_ = *2t*_*0*_. The leg lengths of weld were set as $w_{0}$ = $w_{1}$ = $\sqrt 2 { }t_{1}$ (in Fig. 17), matching those of specimens in tests, where *t*_*1*_ and *t*_*0*_ are brace and chord thickness, respectively.. The cross-section of weld in the model was simplified and simulated as an equilateral right triangle, which is shown in Fig. 18.

According to the research of Peter Balty^37^ and Xu^38^, the friction coefficient between core concrete and steel tube was commonly between 0.2 to 0.6, the study of Wang^39^ showed that the frictional coefficient has little influence on SCFs. In the FE models of this paper, a frictional coefficient of 0.3 was adopted between concrete and steel.

**Figure 19:**
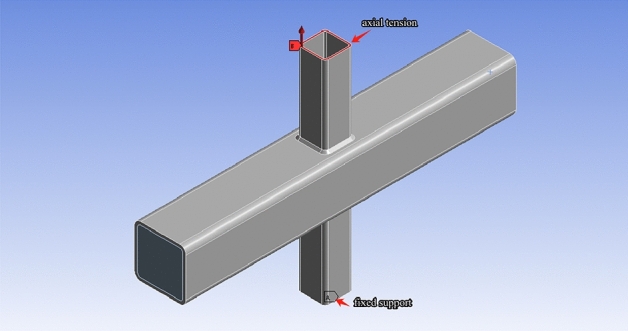
The boundary condition in axial tension FE models.

In Fig. 19, boundary condition in the FE models was defined according to the experimental tests, with fixed support on one end of the brace, and axial tension on the other brace, simultaneously. The dimensions of the finite element models basically followed those of the specimens tested in this paper, adhering to the same equations and patterns.

### Model verification

**Figure 20:**
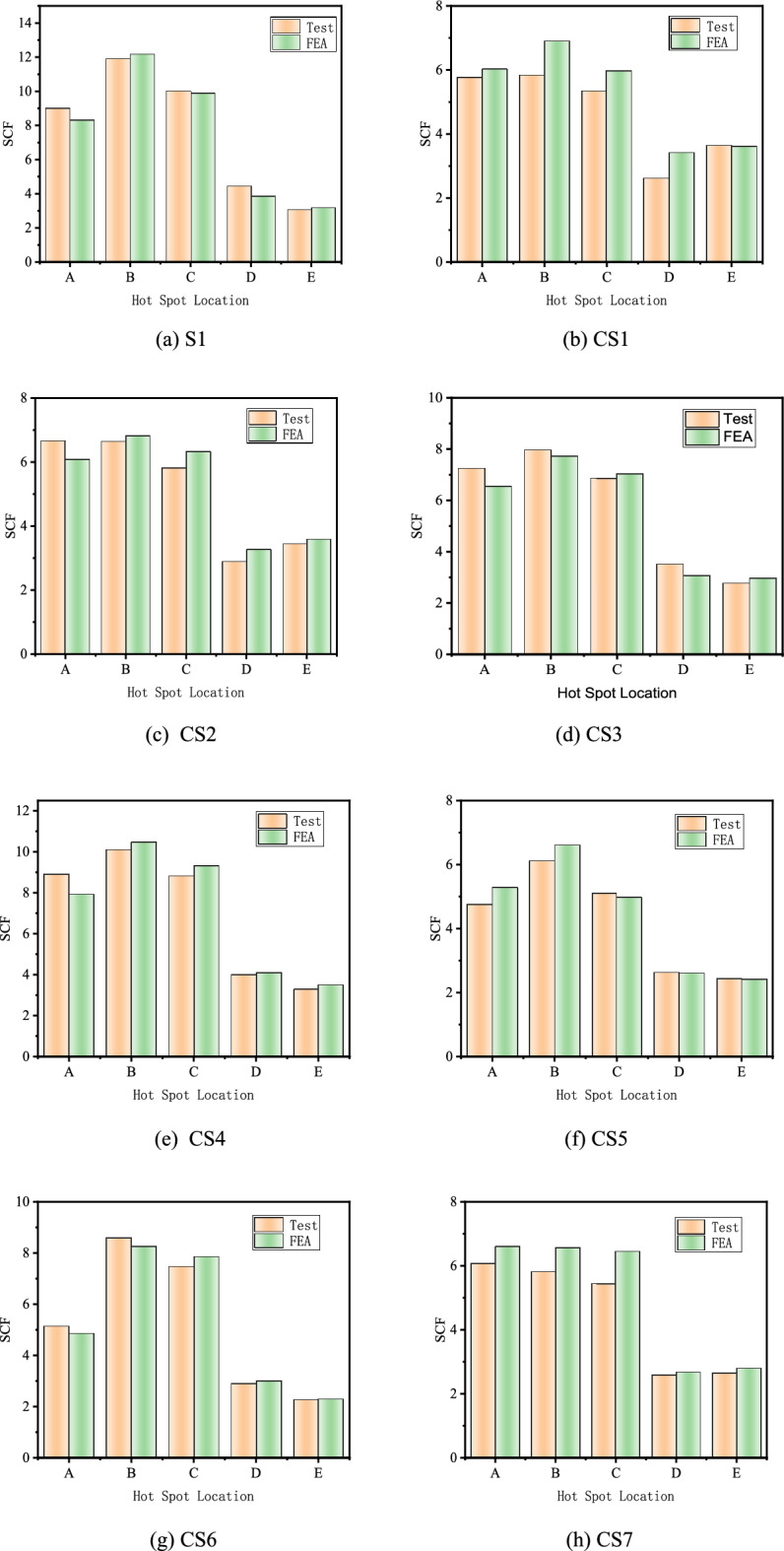
Comparison between experimental results and FE analysis.

The stress values of potential hot spot locations(lines $A$, $B$, $C$, $D,$ and $E$), corresponding to the locations of experimental strain gauge placement, were obtained in FE models. Subsequently, the values of SCFs at lines $A$, $B$, $C$, $D$ and $E$ in the FE models were compared with the ones of SCFs in corresponding specimens of the test, as depicted in Fig. 20 and Table 3. $SCF_{{{\text{test}}}}$ represents the SCF of the test, and $SCF_{{{\text{FE}}}}$ represents the SCF of FE models.

**Table 3:** Comparison of SCFs between FE and Test.

Specimens	SCF result	Locations						
		A	B	C	D	E	Mean	SD
S1	$SCF_{{{\text{test}}}}$	8.98	11.88	9.98	4.43	3.05	–	–
	$SCF_{{{\text{FE}}}}$	8.29	12.14	9.86	3.84	3.16	–	–
	$SCF_{{{\text{test}}}}$/$SCF_{{{\text{FE}}}}$	1.08	0.98	1.01	1.15	0.97	1.04	0.071
CS1	$SCF_{{{\text{test}}}}$	5.75	5.82	5.33	2.61	3.63	–	–
	$SCF_{{{\text{FE}}}}$	6.01	6.89	5.96	3.41	3.61	–	–
	$SCF_{{{\text{test}}}}$/$SCF_{{{\text{FE}}}}$	0.96	0.85	0.90	0.77	1.01	0.89	0.084
CS2	$SCF_{{{\text{test}}}}$	6.66	6.64	5.82	2.91	3.45	–	–
	$SCF_{{{\text{FE}}}}$	6.08	6.81	6.33	3.28	3.60	–	–
	$SCF_{{{\text{test}}}}$/$SCF_{{{\text{FE}}}}$	1.10	0.98	0.92	0.89	0.96	0.97	0.071
CS3	$SCF_{{{\text{test}}}}$	7.25	7.96	6.87	3.50	2.76	–	–
	$SCF_{{{\text{FE}}}}$	6.55	7.72	7.05	3.05	2.94	–	–
	$SCF_{{{\text{test}}}}$/$SCF_{{{\text{FE}}}}$	1.11	1.03	0.97	1.15	0.94	1.04	0.078
CS4	$SCF_{{{\text{test}}}}$	8.93	10.12	8.84	4.00	3.28	–	–
	$SCF_{{{\text{FE}}}}$	7.91	10.49	9.34	4.09	3.50	–	–
	$SCF_{{{\text{test}}}}$/$SCF_{{{\text{FE}}}}$	1.13	0.96	0.95	0.98	0.94	0.99	0.07
CS5	$SCF_{{{\text{test}}}}$	4.76	6.13	5.10	2.64	2.43	–	–
	$SCF_{{{\text{FE}}}}$	5.28	6.63	4.98	2.61	2.41	–	–
	$SCF_{{{\text{test}}}}$/$SCF_{{{\text{FE}}}}$	0.90	0.93	1.03	1.01	1.01	0.97	0.051
CS6	$SCF_{{{\text{test}}}}$	5.15	8.58	7.46	2.91	2.27	–	–
	$SCF_{{{\text{FE}}}}$	4.86	8.25	7.86	3.00	2.30	–	–
	$SCF_{{{\text{test}}}}$/$SCF_{{{\text{FE}}}}$	1.06	1.04	0.95	0.97	0.99	1.00	0.042
CS7	$SCF_{{{\text{test}}}}$	6.07	5.82	5.45	2.59	2.65	–	–
	$SCF_{{{\text{FE}}}}$	6.60	6.56	6.45	2.68	2.80	–	–
	$SCF_{{{\text{test}}}}$/$SCF_{{{\text{FE}}}}$	0.92	0.88	0.84	0.97	0.95	0.91	0.043

**Figure 21:**
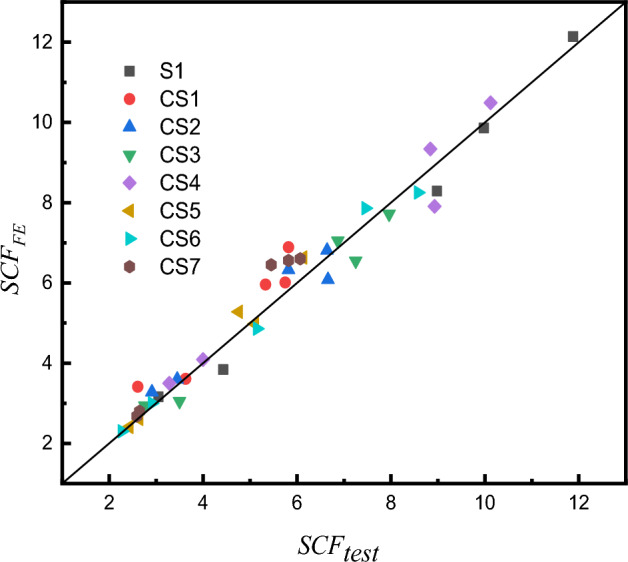
Comparison of SCFs between FE analysis and test results.

According to Figs. 20, 21, the stress distribution pattern in FE models is consistent with that in the test, the maximum SCF of the brace always occurs at the line A, and the maximum SCF of chord occurs at the line B.

As shown in Table 3, the average values of $SCF_{{{\text{test}}}}$/$SCF_{{{\text{FE}}}}$ are from 0.89 to 1.04, the standard deviations are from 0.042 to 0.084. It indicates that FE models can simulate the specimens and predict the SCFs of X-joints well.

### Parametric studies

#### General

As the simulation of the FE models for X-joint had been proved to be practicable according to the aforementioned analysis in this paper, 64 FE models of SHS-CFSHS X-joints with larger parameter ranges were built to make a further study about the effects of three non-dimensional parameters (β, τ and 2γ) on the SCFs. The details of the 64 FE models are listed in Tables 4 and 5, the name of the FE models represents the section sizes of the chord and brace in X-joint, for example, the C110X8.8-B44X2.2 represents that the width and thickness of the chord tube are 110 mm and 8.8 mm, respectively, and the width and thickness of the brace tube are 44 mm and 2.2 mm, respectively. SCF_FE_ is the SCF obtained by FE analysis. According to CIDECT Guide No. 8^10^, The ranges that non-dimensional parameters varied within are as follows:

**Table 4:** SCFs obtained by FE analysis(SCF_FE_) for X-joints under axial tension(AT).

FE models	Non-dimensional parameters			SCF_FE_				
	β	2γ	τ	A	B	C	D	E
C110X8.8-B44X2.2	0.40	12.5	0.25	3.41	2.25	1.96	1.42	2.72
C110X8.8-B44X4.4	0.40	12.5	0.50	3.67	3.09	2.96	1.66	2.36
C110X8.8-B44X6.6	0.40	12.5	0.75	3.69	3.55	3.34	2.22	1.83
C110X8.8-B44X8.8	0.40	12.5	1.00	3.82	6.85	6.07	2.61	1.46
C110X6.88-B44X1.72	0.40	16.0	0.25	5.05	3.25	2.88	2.22	3.95
C110X6.88-B44X3.44	0.40	16.0	0.50	5.26	4.29	4.08	2.35	3.14
C110X6.88-B44X5.16	0.40	16.0	0.75	5.54	6.45	5.04	2.57	2.73
C110X6.88-B44X6.88	0.40	16.0	1.00	4.87	6.92	6.52	3.14	2.00
C110X5.5-B44X1.38	0.40	20.0	0.25	6.41	4.40	4.00	2.81	5.07
C110X5.5-B44X2.75	0.40	20.0	0.50	6.99	6.36	6.05	3.16	4.94
C110X5.5-B44X4.13	0.40	20.0	0.75	7.08	7.42	6.25	3.40	3.73
C110X5.5-B44X5.5	0.40	20.0	1.00	7.25	9.63	7.32	3.87	3.12
C110X4.4-B44X1.1	0.40	25.0	0.25	8.23	6.42	5.98	3.97	7.18
C110X4.4-B44X2.2	0.40	25.0	0.50	8.88	8.90	7.79	4.99	6.41
C110X4.4-B44X3.3	0.40	25.0	0.75	9.18	11.63	10.98	5.33	5.38
C110X4.4-B44X4.4	0.40	25.0	1.00	8.61	13.19	11.63	5.69	4.21
C110X8.8-B60X2.2	0.55	12.5	0.25	3.60	2.05	1.72	1.20	3.03
C110X8.8-B60X4.4	0.55	12.5	0.50	4.11	2.55	2.33	1.45	2.69
C110X8.8-B60X6.6	0.55	12.5	0.75	4.51	3.15	2.65	1.69	2.37
C110X8.8-B60X8.8	0.55	12.5	1.00	4.70	5.53	4.22	2.32	1.78
C110X6.88-B60X1.72	0.55	16.0	0.25	5.73	2.97	2.46	1.88	4.13
C110X6.88-B60X3.44	0.55	16.0	0.50	6.13	4.08	3.74	2.12	3.81
C110X6.88-B60X5.16	0.55	16.0	0.75	6.17	4.68	4.19	2.37	3.15
C110X6.88-B60X6.88	0.55	16.0	1.00	6.04	6.16	5.31	2.98	2.82
C110X5.5-B60X1.38	0.55	20.0	0.25	6.93	4.16	3.70	2.57	6.54
C110X5.5-B60X2.75	0.55	20.0	0.50	7.55	6.06	5.38	2.93	5.06
C110X5.5-B60X4.13	0.55	20.0	0.75	7.76	6.94	6.00	3.14	4.22
C110X5.5-B60X5.5	0.55	20.0	1.00	8.24	7.42	6.43	3.60	4.05
C110X4.4-B60X1.1	0.55	25.0	0.25	8.92	5.60	5.10	3.59	7.76
C110X4.4-B60X2.2	0.55	25.0	0.50	10.19	8.35	7.48	4.26	7.03
C110X4.4-B60X3.3	0.55	25.0	0.75	10.85	10.64	9.33	4.62	6.03
C110X4.4-B60X4.4	0.55	25.0	1.00	11.26	12.07	10.59	4.59	4.63
C110X8.8-B77X2.2	0.70	12.5	0.25	3.35	1.41	1.25	1.03	2.53
C110X8.8-B77X4.4	0.70	12.5	0.50	3.72	2.08	1.62	1.23	2.48
C110X8.8-B77X6.6	0.70	12.5	0.75	4.40	2.71	2.33	1.46	2.13
C110X8.8-B77X8.8	0.70	12.5	1.00	4.59	4.27	4.05	1.96	1.90
C110X6.88-B77X1.72	0.70	16.0	0.25	3.83	2.06	1.67	1.31	3.33
C110X6.88-B77X3.44	0.70	16.0	0.50	5.22	2.73	2.36	1.50	3.17
C110X6.88-B77X5.16	0.70	16.0	0.75	5.66	3.17	2.61	1.92	2.86
C110X6.88-B77X6.88	0.70	16.0	1.00	5.71	5.00	4.37	2.54	2.52
C110X5.5-B77X1.38	0.70	20.0	0.25	5.19	2.97	2.38	1.87	4.48
C110X5.5-B77X2.75	0.70	20.0	0.50	6.33	4.22	3.79	2.16	4.06
C110X5.5-B77X4.13	0.70	20.0	0.75	7.06	4.55	4.08	2.42	3.58
C110X5.5-B77X5.5	0.70	20.0	1.00	7.47	5.61	4.70	3.04	3.12
C110X4.4-B77X1.1	0.70	25.0	0.25	6.89	3.95	3.35	2.73	5.64
C110X4.4-B77X2.2	0.70	25.0	0.50	8.93	6.63	5.58	3.32	5.67
C110X4.4-B77X3.3	0.70	25.0	0.75	9.52	7.20	6.22	3.65	4.79
C110X4.4-B77X4.4	0.70	25.0	1.00	10.00	8.24	7.39	3.89	4.26
C110X8.8-B93X2.2	0.85	12.5	0.25	2.54	1.12	0.87	0.76	2.11
C110X8.8-B93X4.4	0.85	12.5	0.50	3.11	1.43	0.99	0.92	2.26
C110X8.8-B93X6.6	0.85	12.5	0.75	3.79	2.55	2.05	1.30	1.98
C110X8.8-B93X8.8	0.85	12.5	1.00	4.04	3.21	2.78	1.77	1.66
C110X6.88-B93X1.72	0.85	16.0	0.25	3.00	1.43	1.10	0.99	2.63
C110X6.88-B93X3.44	0.85	16.0	0.50	4.00	2.18	1.58	1.31	2.53
C110X6.88-B93X5.16	0.85	16.0	0.75	4.97	2.90	2.33	1.75	2.33
C110X6.88-B93X6.88	0.85	16.0	1.00	5.29	4.14	3.68	2.04	2.14
C110X5.5-B93X1.38	0.85	20.0	0.25	3.55	2.08	1.57	1.27	3.31
C110X5.5-B93X2.75	0.85	20.0	0.50	4.54	3.23	2.54	1.44	3.16
C110X5.5-B93X4.13	0.85	20.0	0.75	5.52	3.58	3.23	2.12	3.11
C110X5.5-B93X5.5	0.85	20.0	1.00	6.24	4.22	3.44	2.05	3.08
C110X4.4-B93X1.1	0.85	25.0	0.25	4.78	2.47	2.00	1.42	3.98
C110X4.4-B93X2.2	0.85	25.0	0.50	5.87	3.32	2.64	1.86	4.07
C110X4.4-B93X3.3	0.85	25.0	0.75	7.02	4.31	3.32	2.41	3.89
C110X4.4-B93X4.4	0.85	25.0	1.00	7.79	5.49	3.52	2.30	3.47

**Table 5:** SCFs obtained by FE analysis(SCF_FE_) for X joints under in-plane bending(IPB).

FE models	Non-dimensional parameters			SCF_FE_				
	β	2γ	τ	A	B	C	D	E
C110X8.8-B44X2.2	0.40	12.5	0.25	1.73	0.91	1.03	0.93	1.71
C110X8.8-B44X4.4	0.40	12.5	0.50	1.6	1.15	1.27	1.28	1.61
C110X8.8-B44X6.6	0.40	12.5	0.75	1.45	1.21	1.3	1.22	1.44
C110X8.8-B44X8.8	0.40	12.5	1.00	1.1	1.41	1.55	1.54	1.22
C110X6.88-B44X1.72	0.40	16.0	0.25	2.52	1.33	1.53	1.37	2.5
C110X6.88-B44X3.44	0.40	16.0	0.50	2.07	1.58	1.75	1.61	2.08
C110X6.88-B44X5.16	0.40	16.0	0.75	1.64	2.04	2.15	1.71	1.64
C110X6.88-B44X6.88	0.40	16.0	1.00	1.51	2.35	2.21	1.89	1.47
C110X5.5-B44X1.38	0.40	20.0	0.25	2.86	1.91	2.28	1.9	2.73
C110X5.5-B44X2.75	0.40	20.0	0.50	2.68	2.4	2.99	2.15	2.52
C110X5.5-B44X4.13	0.40	20.0	0.75	2.35	2.76	3.27	2.71	2.15
C110X5.5-B44X5.5	0.40	20.0	1.00	2.07	2.45	3.16	2.38	1.78
C110X4.4-B44X1.1	0.40	25.0	0.25	3.88	2.34	2.62	2.36	3.99
C110X4.4-B44X2.2	0.40	25.0	0.50	3.57	3.66	4.06	3.3	3.5
C110X4.4-B44X3.3	0.40	25.0	0.75	2.6	4.20	4.15	3.48	2.58
C110X4.4-B44X4.4	0.40	25.0	1.00	2.88	5.85	4.9	4.2	2.72
C110X8.8-B60X2.2	0.55	12.5	0.25	2.24	1.11	1.29	1.09	2.03
C110X8.8-B60X4.4	0.55	12.5	0.50	2.43	1.4	1.41	1.36	2.17
C110X8.8-B60X6.6	0.55	12.5	0.75	2.22	1.35	1.6	1.43	2.09
C110X8.8-B60X8.8	0.55	12.5	1.00	2.09	2.45	2.65	2.24	1.76
C110X6.88-B60X1.72	0.55	16.0	0.25	3.17	1.79	2.17	1.77	3.06
C110X6.88-B60X3.44	0.55	16.0	0.50	2.89	2.41	2.41	2.06	2.77
C110X6.88-B60X5.16	0.55	16.0	0.75	2.91	3.07	3.04	2.84	2.66
C110X6.88-B60X6.88	0.55	16.0	1.00	2.58	3.85	3.49	2.69	2.4
C110X5.5-B60X1.38	0.55	20.0	0.25	4.28	2.22	2.35	2.02	4.31
C110X5.5-B60X2.75	0.55	20.0	0.50	3.72	3.22	3.52	2.8	3.42
C110X5.5-B60X4.13	0.55	20.0	0.75	3.42	3.42	3.58	2.75	2.99
C110X5.5-B60X5.5	0.55	20.0	1.00	3.39	4.47	4.47	3.04	2.88
C110X4.4-B60X1.1	0.55	25.0	0.25	6.17	3.87	4.24	3.62	6.39
C110X4.4-B60X2.2	0.55	25.0	0.50	5.09	5.77	6.62	5.07	4.77
C110X4.4-B60X3.3	0.55	25.0	0.75	5.05	7.38	6.74	4.78	4.43
C110X4.4-B60X4.4	0.55	25.0	1.00	4.64	9.05	7.67	5.57	3.76
C110X8.8-B77X2.2	0.70	12.5	0.25	2.31	1.04	1.23	0.93	2.41
C110X8.8-B77X4.4	0.70	12.5	0.50	2.93	1.46	1.78	1.41	2.37
C110X8.8-B77X6.6	0.70	12.5	0.75	3.17	1.45	1.87	1.46	2.43
C110X8.8-B77X8.8	0.70	12.5	1.00	2.74	2.76	2.87	2.75	2.6
C110X6.88-B77X1.72	0.70	16.0	0.25	2.99	1.69	1.68	1.45	2.96
C110X6.88-B77X3.44	0.70	16.0	0.50	4.08	3.54	3.53	2.63	3.5
C110X6.88-B77X5.16	0.70	16.0	0.75	4.28	4.25	3.92	3.02	4.06
C110X6.88-B77X6.88	0.70	16.0	1.00	3.74	4.82	4.96	3.46	3.43
C110X5.5-B77X1.38	0.70	20.0	0.25	3.84	2.28	2.46	1.64	3.99
C110X5.5-B77X2.75	0.70	20.0	0.50	4.51	4.04	3.73	2.84	4.02
C110X5.5-B77X4.13	0.70	20.0	0.75	4.55	3.82	3.58	2.64	3.45
C110X5.5-B77X5.5	0.70	20.0	1.00	3.71	3.9	3.57	2.74	3.52
C110X4.4-B77X1.1	0.70	25.0	0.25	7.94	5.78	6.52	5.02	7.64
C110X4.4-B77X2.2	0.70	25.0	0.50	5.85	4.96	6.08	4.81	5.44
C110X4.4-B77X3.3	0.70	25.0	0.75	5.83	8.44	7.62	5.37	5.01
C110X4.4-B77X4.4	0.70	25.0	1.00	5.89	10	9.42	5.62	4.8
C110X8.8-B93X2.2	0.85	12.5	0.25	2.11	1.09	1	1.01	2.27
C110X8.8-B93X4.4	0.85	12.5	0.50	2.92	1.37	1.62	1.36	2.7
C110X8.8-B93X6.6	0.85	12.5	0.75	3.26	2.01	1.96	1.76	3.23
C110X8.8-B93X8.8	0.85	12.5	1.00	3.29	2.13	2.64	1.81	3.23
C110X6.88-B93X1.72	0.85	16.0	0.25	4.19	2.77	2.95	2.17	3.92
C110X6.88-B93X3.44	0.85	16.0	0.50	4.17	4.13	3.73	2.47	3.63
C110X6.88-B93X5.16	0.85	16.0	0.75	4.84	4.56	3.33	2.78	3.95
C110X6.88-B93X6.88	0.85	16.0	1.00	4.15	5.08	3.9	4.06	3.94
C110X5.5-B93X1.38	0.85	20.0	0.25	4.31	2.1	2.43	1.93	4.12
C110X5.5-B93X2.75	0.85	20.0	0.50	4.78	4.37	3.46	2	4.53
C110X5.5-B93X4.13	0.85	20.0	0.75	5.05	4.18	3.84	1.98	4.48
C110X5.5-B93X5.5	0.85	20.0	1.00	5.35	3.85	4.24	2.81	5.05
C110X4.4-B93X1.1	0.85	25.0	0.25	3.44	1.42	2.00	1.29	3.58
C110X4.4-B93X2.2	0.85	25.0	0.50	6.31	2.97	2.72	2.79	6.24
C110X4.4-B93X3.3	0.85	25.0	0.75	6.65	3.58	3.54	3.27	6.67
C110X4.4-B93X4.4	0.85	25.0	1.00	6.57	3.57	3.89	4.25	7.36

0.4 ≤ β ≤ 0.85.

12.5 ≤ 2γ ≤ 25.0,

0.25 ≤ τ ≤ 1.00.

**Figure 22:**
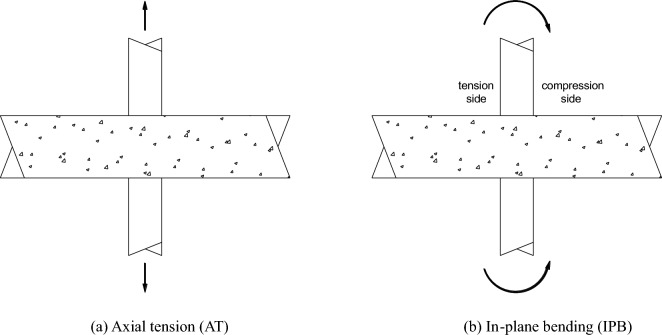
Load conditions.

Two load conditions were considered, and they were axial tension in the brace (AT) and in-plane bending in the brace (IPB), which is shown in Fig. 22. Lei et al.^23^ and Lin et al.^40^ studied SCFs of the tubular joints with brace under axial tension, and Zheng et al.^14^ and Tong et al.^16^ found that When the brace of the T-joint was under tension, the SCFs in the joint were much higher than those under compression.

According to the FE analysis, when the braces are subjected to the axial load, the values of SCFs determined under axial tension load are much larger than the ones under axial compression load; when the braces are subjected to the in-plane bending load, the values of SCFs at the tension side are much larger than the ones at the compression side; Compared to compressive force, it is no doubt that tensile force is more adverse to a structure’s fatigue life. Therefore, the SCFs under the AT load condition were studied (Fig. 22a) and the SCFs at the tension side were studied for the IPB load condition (Fig. 22b).

#### AT load condition

The results of FE analysis about the X-joints under AT load condition are listed in Table 4, which shows the SCFs at the line A, B, C, D and E in every FE model.

(1) Effect of $$\beta$$

**Figure 23:**
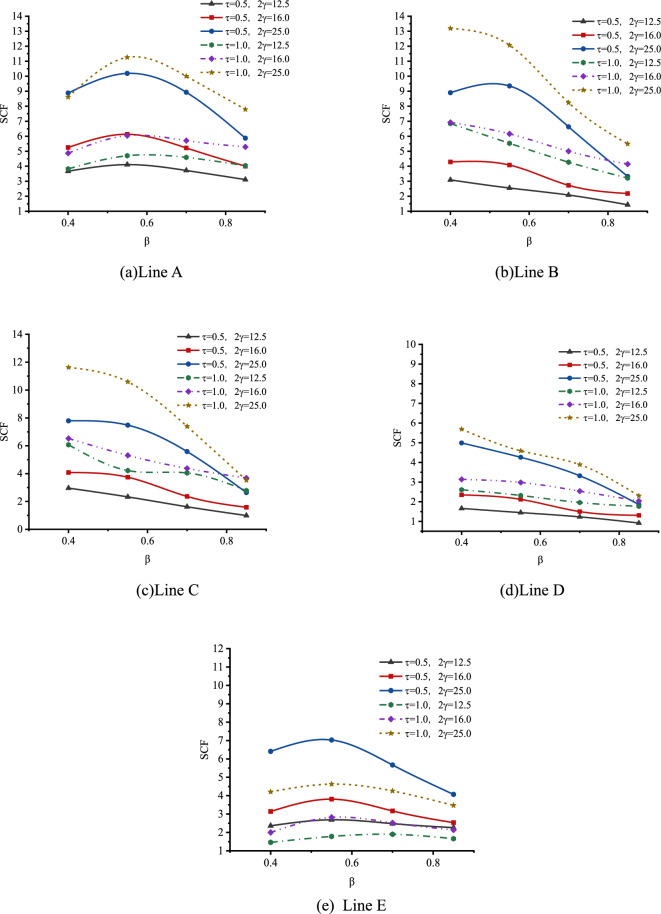
Effect of β on SCFs.

The effect of β on SCFs of SHS-CFSHS X-joint is analyzed, and the change patterns of SCFs as β increases at the lines of A, B, C, D and E are obtained, which is illustrated in Fig. 23.

As shown in Fig. 23, for both line A and line E, the values of SCFs increase first and then decrease, as β increases from 0.4 to 0.85. For lines B, C, and D, the values of SCFs decrease with β increasing. The highest SCFs of line A and E occur when β is approximately 0.55, which is consistent with Ref.^10^. The shapes of the curves for lines A, B, C, D, and E in SHS-CFSHS X-joints are similar to the ones in the corresponding X-joints without filling concrete in CIDECT^10^, but all of the values of SCFs are lower than the ones in Ref^10^. due to the filling of concrete.

(2) Effect of 2γ

**Figure 24:**
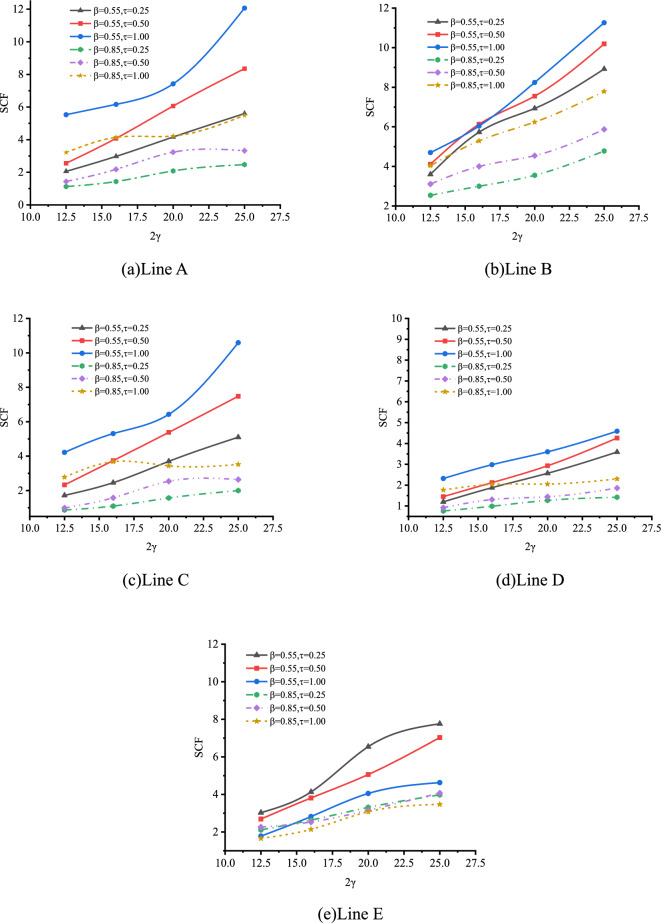
Effect of 2γ on SCFs.

The effect of 2γ on SCFs of SHS-CFSHS X-joint is investigated by FE method, and the change patterns of SCFs are obtained as β increases at the location of line A, B, C, D and E(in Fig. 24).

As shown in Fig. 24, the values of SCFs increase with the 2γ increasing from 12.5 to 25 for lines A, B, C, D and E. The maximum SCFs of brace and chord occur at lines A and B respectively. When 2γ increases, the thickness of the chord becomes thinner, which leads to a larger bending deformation in the steel plate of the chord as the brace is under the same degree of tension, and results in higher SCFs.

(3) Effect of τ

**Figure 25:**
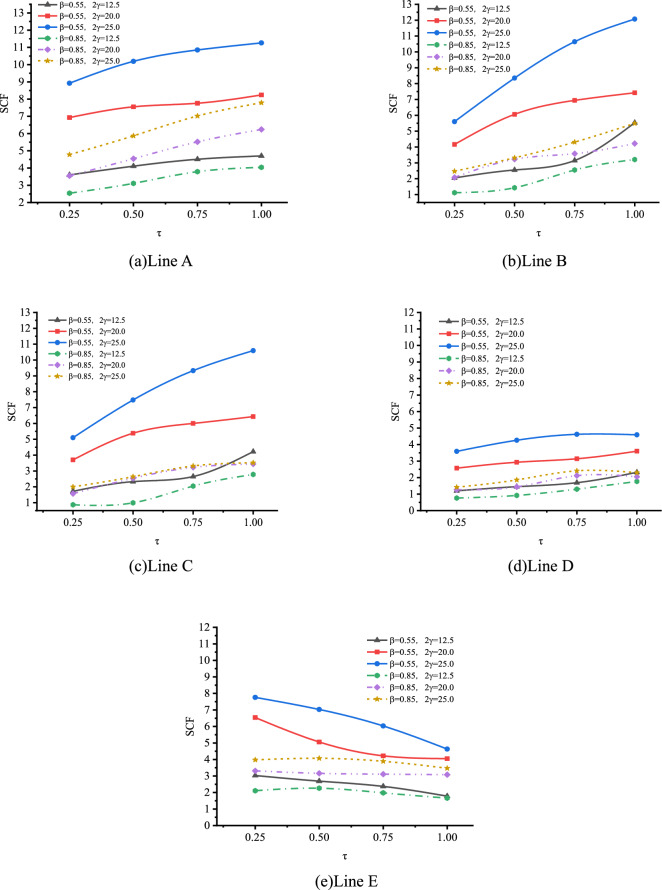
Effects of τ on SCFs.

The effect of τ on SCFs of SHS-CFSHS X-joint is investigated, and the change patterns of SCFs at lines A, B, C, D and E are obtained asτ increases(in Fig. 25). For lines A, B, C and D, SCFs increases as τ increases, and SCFs decrease as τ increases for line E. The maximum SCFs on brace and chord always occur at lines A and B, respectively.

#### IPB load condition

**Figure 26:**
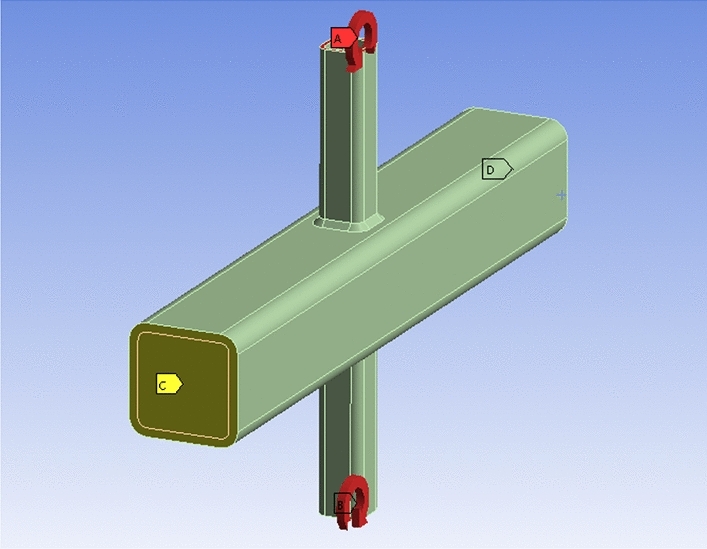
FE model under in-plane bending.

In Fig. 26, FE models under in-plane bending, each side of chord is set with a “remote displacement” boundary condition, simulating a hinge joint in practical applications, and each side of brace is set with “moment”, simulating bending moment in practical applications.

The results of FE analysis about the X-joints under IPB load condition are listed in Table 5, which shows the SCFs at the line A, B, C, D and E in every FE model.

(1) Effect of $$\beta$$

**Figure 27:**
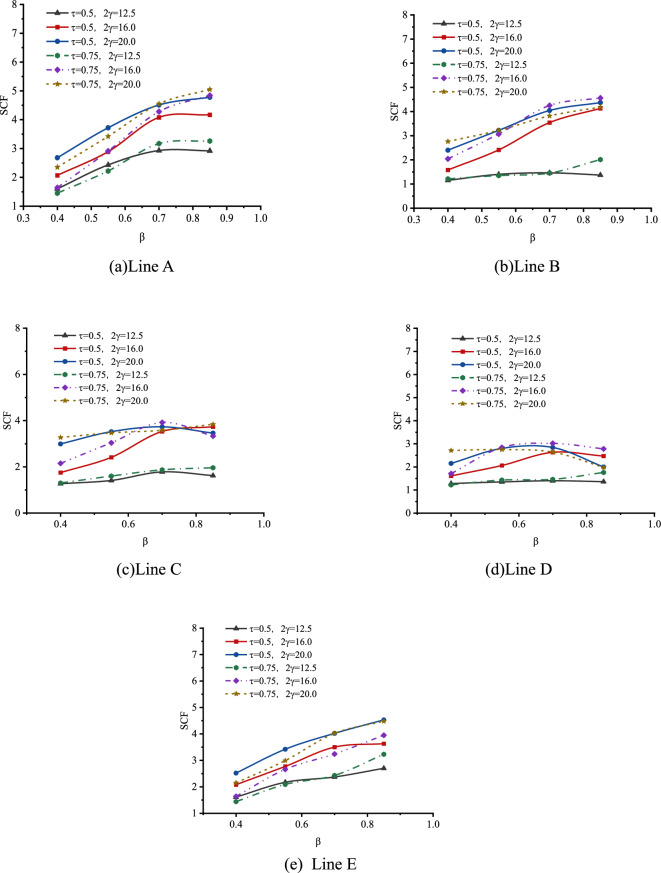
Effect of β on SCFs.

The effect of β on SCFs of SHS-CFSHS X-joint is analyzed, and the change patterns of SCFs as β increases at the lines of A, B, C, D and E are obtained, which is illustrated in Fig. 27.

As shown in Fig. 27, for lines A, B, and E, the values of SCFs increase as β increases from 0.4 to 0.85; in addition, after reaching 0.7, there is a gradual deceleration in the upward trend of the SCFs. For lines C and D, the upward trends of SCFs are not so significant compared with lines A, B and E, and the values of SCFs are in the range of 1 to 4. The highest SCFs of lines A, B, and E occur when β is approximately 0.7 or 0.85, which is similar with the highest SCFs of lines A, B, and E of hollow X-joints in Ref.^10^, but the difference is the values of SCFs of SHS-CFSHS X-joints are lower than the ones of SCFs of corresponding hollow X-joints due to the filling of concrete.

(2) Effect of 2γ

**Figure 28:**
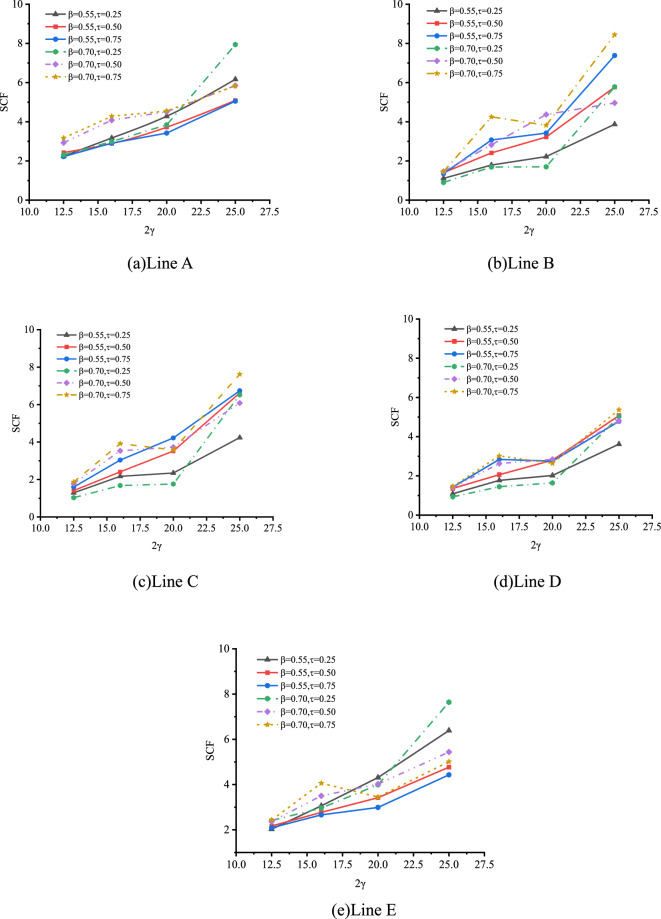
Effect of 2γ on SCFs.

The effect of 2γ on SCFs of SHS-CFSHS X-joint is investigated by FE method, and the change patterns of SCFs are obtained as 2γ increases at the location of line A, B, C, D and E(in Fig. 28).

As shown in Fig. 28, the values of SCFs increase with the 2γ increasing from 12.5 to 25 for lines A, B, C, D and E. The maximum SCFs of brace and chord occur at lines A and B respectively. When 2γ increases, the thickness of the chord becomes thinner, which leads to a larger bending deformation in the steel plate of the chord as the brace is under the same load, and results in higher SCFs.

(3) Effect of τ

**Figure 29:**
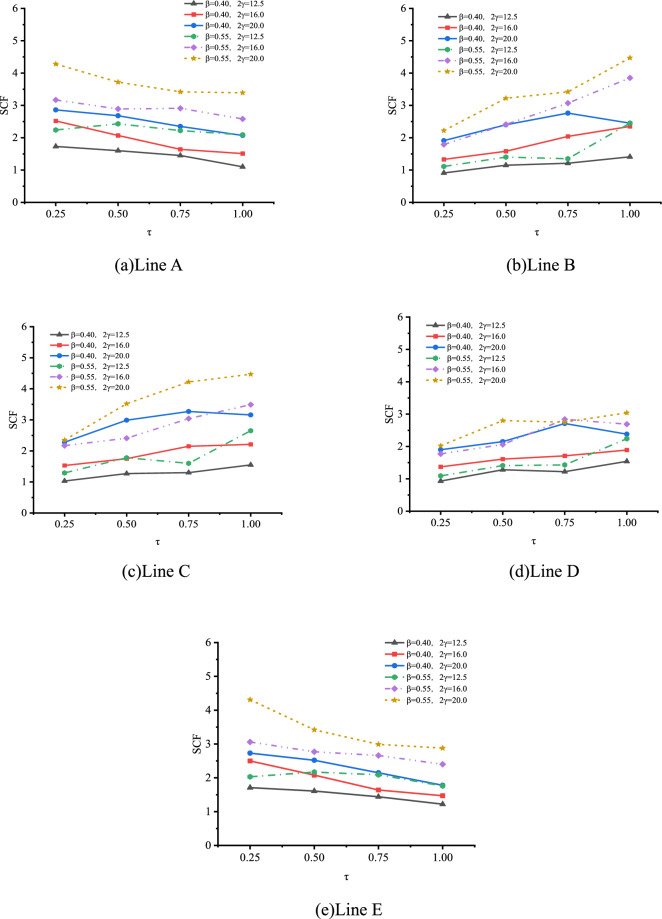
Effects of τ on SCFs.

The effect of τ on SCFs of SHS-CFSHS X-joint is investigated, and the change patterns of SCFs at lines A, B, C, D and E are obtained as τ increases(in Fig. 29). For lines A and E, the SCFs decreases slightly as τ increases and the curves drops gently. In addition, the values of SCF in line A and line E are nearly identical, which is in accordance with the situation of hollow X-joints in Ref.^10^. For lines B, C, and D, the SCFs increases slightly as τ increases.

#### *Comparison with Ref*^23^

**Figure 30:**
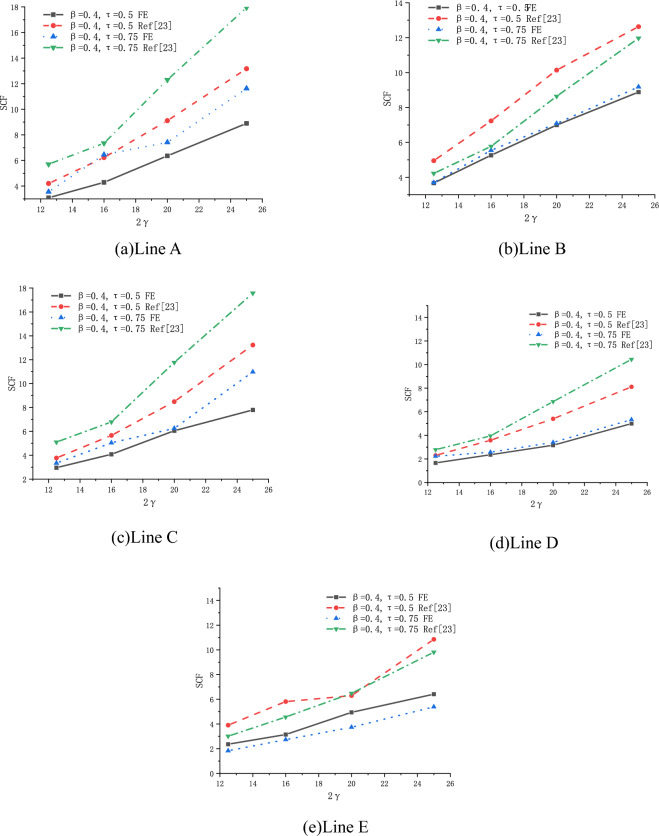
Effects of 2γ on SCFs.

**Figure 31:**
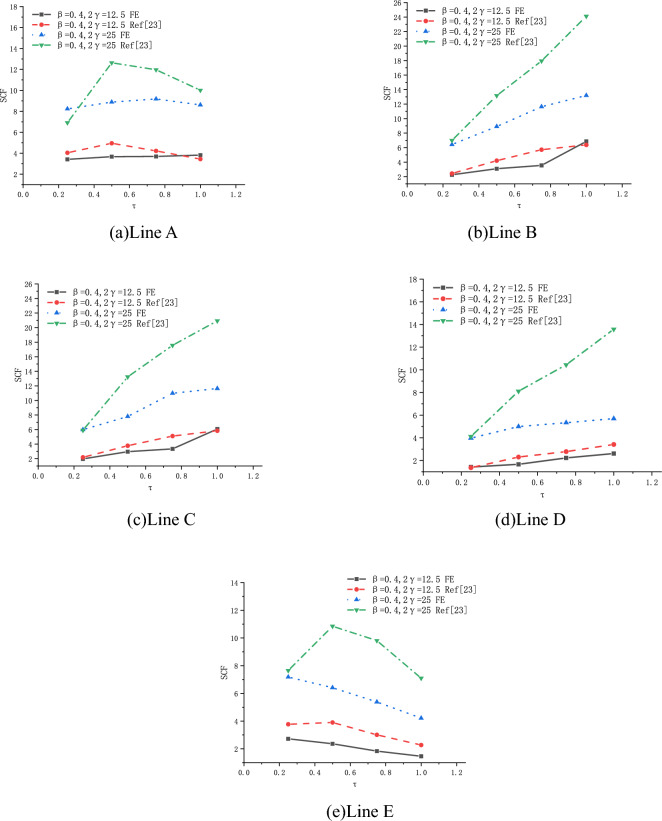
Effects of τ on SCFs.

**Figure 32:**
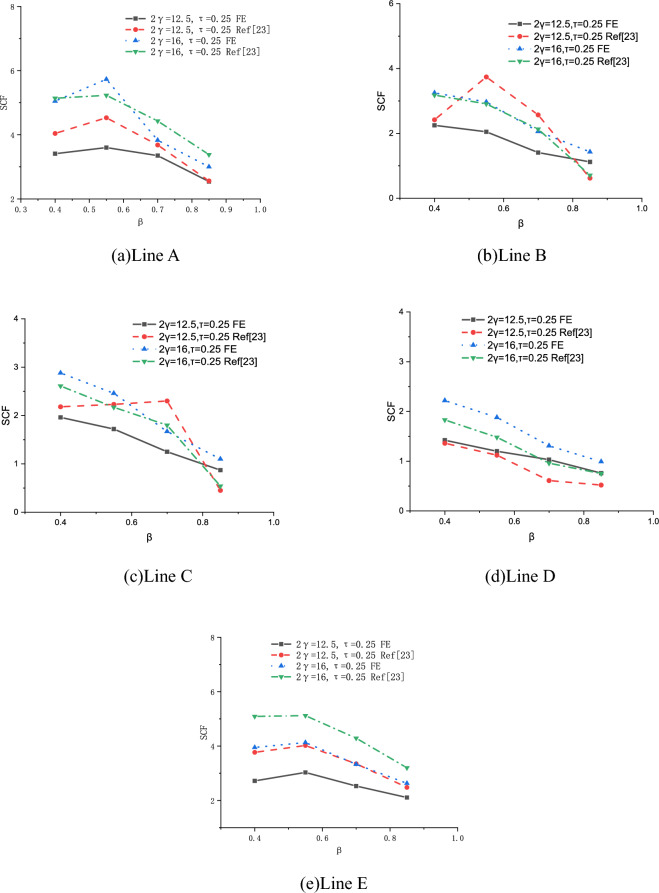
Effects of β on SCFs.

In Ref^23^, FE models of concrete-filled steel tube X-joints were built, and the effects of $\beta$, 2γ and τ on SCFs were investigated. In this section, comparison between this study and Ref^23^ is conducted, as shown in Figs. 30, 31, 32. The overall change pattern and distributional pattern of SCFs are basically consistent. Figures 30, 31, 32 also show that SCFs of Ref^23^ are higher than those of this study, the reasons may be because of the cold-formed angles of the steel tube, which is ${\text{R}}_{0}$ for chord and ${\text{R}}_{1}$ for brace in Fig. 1b, c. In FE models of Ref^23^, the cold formed angles were disregarded. However, in this study, cold-formed angles are considered, which cause smooth transition of edges, consequently leading to the lower SCFs than Ref^23^.

## Proposed SCF formulae and verification

### SCF formulae

According to Ref^10,23^, the general format of SCF formulae in this paper is proposed as follows(formula 2):

2 $$SCF = (c_{1} + c_{2} \beta + c_{3} \beta^{2} + c_{4} \cdot 2\gamma ) \cdot (2\gamma )^{{(c_{5} + c_{6} \beta + c_{7} \beta^{2} )}} \cdot \tau^{{(c_{8} + c_{9} \beta )}}$$

where $c_{1}$ to $c_{9}$ are constants and obtained by regression analysis.

Based on the results of FE analysis, multiple regression analysis was used to obtain the SCF formulae of SHS-CFSHS X-joints under axial tension in the brace and in-plane bending in the brace. The formulae(3)–(7) are proposed for predicting the SCFs of SHS-CFSHS X-joints under axial tension in the brace load condition:

Line A:

3 $$SCF = (0.59 - 2.16\beta + 2.921\beta^{2} + 0.106 \cdot 2\gamma ) \cdot (2\gamma )^{{( - 0.177 + 2.107\beta - 1.898\beta^{2} )}} \cdot \tau^{( - 0.274 + 0.758\beta )}$$

Line B:

4 $$SCF = (1.629 - 4.985\beta + 4.272\beta^{2} + 0.022 \cdot 2\gamma ) \cdot (2\gamma )^{{(0.196 + 2.719\beta - 2.772\beta^{2} )}} \cdot \tau^{(0.537 + 0.0036\beta )}$$

Line C:

5 $$SCF = (1.789 - 5.981\beta + 5.345\beta^{2} + 0.0238 \cdot 2\gamma ) \cdot (2\gamma )^{{( - 0.008 + 3.449\beta - 3.512\beta^{2} )}} \cdot \tau^{(0.4104 + 0.195\beta )}$$

Line D:

6 $$SCF = (0.467 - 1.503\beta + 1.564\beta^{2} + 0.0079 \cdot 2\gamma ) \cdot (2\gamma )^{{(0.469 + 1.923\beta - 2.223\beta^{2} )}} \cdot \tau^{(0.0204 + 0.446\beta )}$$

Line E:

7 $$SCF = (0.021 - 0.151\beta + 0.366\beta^{2} - 0.000235 \cdot 2\gamma ) \cdot (2\gamma )^{{(2.793 - 2.802\beta + 0.79\beta^{2} )}} \cdot \tau^{( - 0.677 + 0.668\beta )}$$

The formulae (8)–(12) were proposed for predicting the SCFs of SHS-CFSHS X-joints under in-plane bending load condition:

Line A:

8 $$SCF = (0.{247} - {0}{\text{.488}}\beta + {0}.{264}\beta^{2} - 0.{00}0{62} \cdot 2\gamma ) \cdot (2\gamma )^{{( - {0}{\text{.0752}} + {3}.{127}\beta - {0}.{639}\beta^{2} )}} \cdot \tau^{{( - 0.{805} + {1}.{163}\beta )}}$$

Line B:

9 $$SCF = ({3}{\text{.002}} - {13}.{021}\beta + {12}.{225}\beta^{2} { + 0}{\text{.0637}} \cdot 2\gamma ) \cdot (2\gamma )^{{( - {1}{\text{.393}} + {6}.{874}\beta - {5}{\text{.845}}\beta^{2} )}} \cdot \tau^{{(0.{692} - 0.{275}\beta )}}$$

Line C:

10 $$SCF = ({2}.{414} - {9}.{038}\beta + {8}{\text{.081}}\beta^{2} { + }0.{0}2{68} \cdot 2\gamma ) \cdot (2\gamma )^{{( - {1}{\text{.518}} + {7}{\text{.836}}\beta - {6}{\text{.552}}\beta^{2} )}} \cdot \tau^{{(0.{353} + 0.{0274}\beta )}}$$

Line D:

11 $$SCF = ({1}.{117} - {4}.{174}\beta + {3}.{921}\beta^{2} { + }0.01{7}5 \cdot 2\gamma ) \cdot (2\gamma )^{{( - {0}{\text{.76 + 5}}{.243}\beta - {4}{\text{.462}}\beta^{2} )}} \cdot \tau^{{(0.1{14} + 0.33\beta )}}$$

Line E:

12 $$SCF = ({0}.{0346} - {0}.0{81}\beta + {0}.{594}\beta^{2} { + }0.0{176} \cdot 2\gamma ) \cdot (2\gamma )^{{( - 0.{1}4{2} + {2}.{005}\beta - 1.{271}\beta^{2} )}} \cdot \tau^{{( - {1}.{055} + 1.{452}\beta )}}$$

### Comparison of SCFs determined from formulae and FE analysis

**Figure 33:**
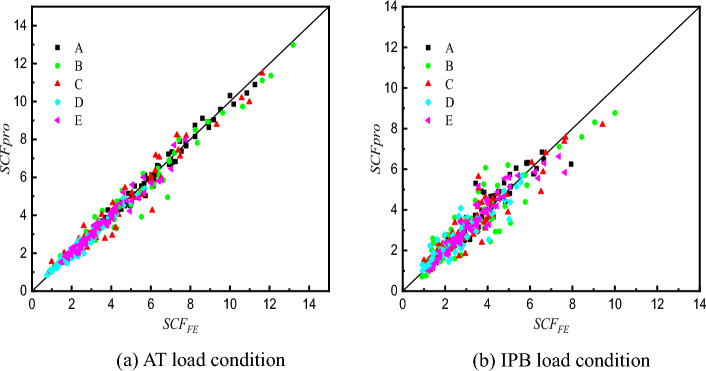
Comparison of SCFs between proposed formulae and FE analysis.

The comparisons of SCFs determined from proposed formulae and FE analysis are listed in Table 6 and shown in Fig. 33 from line A to line E in the X-joints. The average values of the ratios of the proposed formulae value ($SCF_{{{\text{pro}}}}$) to the FE analysis value ($SCF_{{{\text{FE}}}}$) are between 0.99 and 1.02 for the AT load condition, and between 1.00 and 1.04 for the IPB load condition. The standard deviations are between 0.045 and 0.135 for the AT load condition, and between 0.119 and 0.264 for the IPB load condition. It can be seen that the values calculated by proposed SCF formulae fit the FE ones quite well. This indicates a good accuracy of the formulae obtained from multiple regression analysis.

**Table 6:** Comparison of SCFs between proposed formulae($SCF_{{{\text{pro}}}}$) and FE analysis($SCF_{{{\text{FE}}}}$).

Specimens	$SCF_{{{\text{pro}}}}/SCF_{{{\text{FE}}}}$									
	AT					IPB				
	A	B	C	D	E	A	B	C	D	E
C110X8.8-B44X2.2	1.07	1.04	1.10	1.07	0.99	1.20	0.78	1.05	1.08	1.13
C110X8.8-B44X4.4	1.01	1.10	1.02	1.05	0.86	1.03	0.92	1.10	0.93	0.87
C110X8.8-B44X6.6	1.02	1.20	1.11	0.85	0.94	0.99	1.11	1.24	1.08	0.80
C110X8.8-B44X8.8	0.99	0.72	0.70	0.77	1.05	1.18	1.12	1.16	0.92	0.82
C110X6.88-B44X1.72	0.98	1.00	1.05	0.96	1.01	1.05	0.83	0.99	1.04	1.04
C110X6.88-B44X3.44	0.96	1.11	1.04	1.04	0.96	1.01	1.05	1.12	1.04	0.90
C110X6.88-B44X5.16	0.92	0.91	1.03	1.03	0.93	1.11	1.03	1.05	1.09	0.94
C110X6.88-B44X6.88	1.06	1.00	0.92	0.89	1.13	1.09	1.05	1.14	1.06	0.91
C110X5.5-B44X1.38	1.02	1.01	1.05	1.04	1.11	1.14	0.85	0.91	1.03	1.25
C110X5.5-B44X2.75	0.96	1.02	0.97	1.07	0.85	0.96	1.01	0.89	1.08	0.97
C110X5.5-B44X4.13	0.96	1.08	1.14	1.07	0.96	0.95	1.11	0.95	0.94	0.94
C110X5.5-B44X5.5	0.94	0.98	1.12	1.00	1.02	0.98	1.48	1.09	1.15	0.99
C110X4.4-B44X1.1	1.06	0.96	0.98	1.03	1.07	1.03	0.99	1.09	1.15	1.13
C110X4.4-B44X2.2	1.00	1.00	1.05	0.94	0.90	0.88	0.95	0.91	0.97	0.93
C110X4.4-B44X3.3	0.98	0.96	0.91	0.95	0.91	1.05	1.05	1.03	1.02	1.04
C110X4.4-B44X4.4	1.06	0.98	0.99	0.95	1.04	0.86	0.89	0.97	0.91	0.86
C110X8.8-B60X2.2	1.03	0.91	0.95	1.06	1.00	1.11	0.68	0.80	0.97	1.17
C110X8.8-B60X4.4	0.99	1.06	1.00	1.05	0.91	0.91	0.79	0.95	0.96	0.92
C110X8.8-B60X6.6	0.96	1.07	1.09	1.01	0.91	0.94	1.02	0.97	1.03	0.86
C110X8.8-B60X8.8	0.96	0.71	0.79	0.79	1.11	0.95	0.65	0.65	0.71	0.95
C110X6.88-B60X1.72	0.88	0.90	0.98	0.94	1.04	1.08	0.82	0.83	0.92	1.04
C110X6.88-B60X3.44	0.91	0.95	0.92	1.01	0.91	1.05	0.89	0.96	0.97	0.96
C110X6.88-B60X5.16	0.95	1.04	1.01	1.00	0.97	0.98	0.87	0.88	0.79	0.90
C110X6.88-B60X6.88	1.01	0.92	0.93	0.86	1.00	1.05	0.81	0.86	0.91	0.93
C110X5.5-B60X1.38	0.96	0.91	0.93	0.95	0.90	1.05	1.11	1.22	1.19	0.97
C110X5.5-B60X2.75	0.98	0.91	0.92	1.00	0.94	1.07	1.11	1.05	1.06	1.02
C110X5.5-B60X4.13	1.01	0.98	1.01	1.04	0.99	1.09	1.30	1.20	1.21	1.06
C110X5.5-B60X5.5	0.99	1.07	1.10	0.98	0.95	1.05	1.16	1.07	1.19	1.02
C110X4.4-B60X1.1	1.00	0.96	0.97	0.94	1.03	0.94	1.01	1.07	0.99	0.87
C110X4.4-B60X2.2	0.97	0.94	0.95	0.95	0.92	1.01	0.99	0.88	0.86	0.98
C110X4.4-B60X3.3	0.96	0.91	0.94	0.98	0.94	0.95	0.96	1.01	1.03	0.95
C110X4.4-B60X4.4	0.97	0.94	0.96	1.06	1.12	0.99	0.92	0.99	0.96	1.04
C110X8.8-B77X2.2	0.97	1.11	1.10	1.07	1.05	1.09	1.32	1.25	1.39	1.07
C110X8.8-B77X4.4	1.04	1.09	1.24	1.13	0.93	0.86	1.33	1.12	1.16	1.06
C110X8.8-B77X6.6	0.98	1.04	1.08	1.09	0.99	0.80	1.63	1.24	1.29	1.02
C110X8.8-B77X8.8	1.01	0.77	0.72	0.89	1.05	0.93	0.99	0.90	0.76	0.94
C110X6.88-B77X1.72	1.12	1.05	1.13	1.11	1.07	1.21	1.24	1.38	1.26	1.15
C110X6.88-B77X3.44	0.98	1.15	1.17	1.22	0.97	0.89	0.83	0.85	0.88	0.95
C110X6.88-B77X5.16	1.00	1.23	1.32	1.09	0.99	0.85	0.85	0.89	0.88	0.81
C110X6.88-B77X6.88	1.07	0.91	0.92	0.91	1.06	0.98	0.87	0.78	0.85	0.94
C110X5.5-B77X1.38	1.07	0.99	1.07	1.01	1.03	1.26	1.33	1.37	1.54	1.11
C110X5.5-B77X2.75	1.05	1.01	0.98	1.10	0.99	1.08	1.06	1.17	1.13	1.07
C110X5.5-B77X4.13	1.04	1.17	1.14	1.12	1.03	1.08	1.38	1.41	1.40	1.23
C110X5.5-B77X5.5	1.06	1.10	1.16	0.98	1.11	1.33	1.56	1.58	1.49	1.19
C110X4.4-B77X1.1	1.05	1.02	1.04	0.91	1.06	0.79	0.76	0.75	0.70	0.76
C110X4.4-B77X2.2	0.97	0.88	0.91	0.94	0.92	1.07	1.25	1.04	0.93	1.05
C110X4.4-B77X3.3	1.01	1.01	1.02	0.98	1.00	1.08	0.90	0.97	0.96	1.12
C110X4.4-B77X4.4	1.03	1.03	1.00	1.01	1.05	1.07	0.88	0.87	1.01	1.15
C110X8.8-B93X2.2	0.95	1.14	1.19	1.11	1.02	1.18	1.35	1.51	1.17	1.07
C110X8.8-B93X4.4	1.00	1.30	1.56	1.21	0.88	0.97	1.47	1.21	1.15	1.02
C110X8.8-B93X6.6	0.96	0.90	0.95	1.01	0.96	0.94	1.21	1.16	1.04	0.92
C110X8.8-B93X8.8	1.00	0.84	0.83	0.83	1.11	0.98	1.30	0.96	1.13	0.97
C110X6.88-B93X1.72	1.00	1.11	1.13	1.03	1.03	0.86	0.64	0.62	0.67	0.80
C110X6.88-B93X3.44	0.97	1.06	1.18	1.03	0.99	0.98	0.59	0.64	0.78	0.98
C110X6.88-B93X5.16	0.91	0.99	1.01	0.90	1.03	0.91	0.64	0.84	0.81	0.97
C110X6.88-B93X6.88	0.95	0.81	0.75	0.87	1.09	1.12	0.66	0.80	0.62	1.02
C110X5.5-B93X1.38	1.04	0.95	0.95	0.96	1.01	1.09	1.02	0.92	0.93	0.97
C110X5.5-B93X2.75	1.05	0.89	0.88	1.12	0.99	1.11	0.67	0.84	1.18	1.00
C110X5.5-B93X4.13	1.00	1.00	0.87	0.89	0.96	1.14	0.84	0.88	1.40	1.09
C110X5.5-B93X5.5	0.99	0.99	0.97	1.03	0.94	1.13	1.05	0.89	1.10	1.02
C110X4.4-B93X1.1	0.96	1.00	0.91	1.04	1.04	1.54	1.83	1.37	1.73	1.45
C110X4.4-B93X2.2	1.01	1.08	1.03	1.05	0.95	0.95	1.20	1.31	1.05	0.94
C110X4.4-B93X3.3	0.98	1.04	1.03	0.95	0.95	0.98	1.20	1.17	1.05	0.94
C110X4.4-B93X4.4	0.98	0.95	1.15	1.12	1.03	1.04	1.37	1.19	0.90	0.90
Average	1.00	1.00	1.02	1.00	0.99	1.03	1.04	1.03	1.04	1.00
Standard Deviation	0.045	0.108	0.135	0.091	0.067	0.124	0.264	0.209	0.204	0.119

By comparing the two graphs in Fig. 33, it can also be found that the maximum SCFs of X-joints under IPB load are smaller than the ones of X-joints under AT load.

### Comparison of SCFs determined from formulae and test

**Figure 34:**
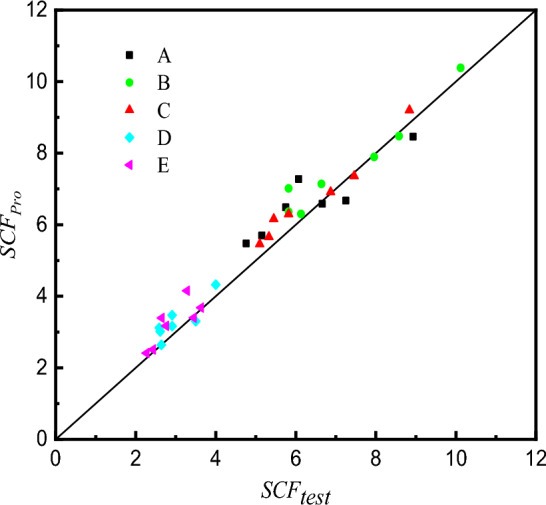
Comparison of SCFs between proposed formulae and test results.

The comparison of SCFs between proposed formulae values and the experimental test values is shown in Table 7 and Fig. 34. The average values of the ratios of the proposed formulae value ($SCF_{{{\text{pro}}}}$) to the FE analysis value ($SCF_{{{\text{test}}}}$) are from 1.05 to 1.11, and the standard deviations are from 0.045 to 0.112.

**Table 7:** Comparison of SCFs between proposed formula ($SCF_{{{\text{pro}}}}$) and test results ($SCF_{{{\text{test}}}}$).

specimens	${\text{ SCF}}_{{{\text{pro}}}}$					${\text{SCF}}_{{{\text{pro}}}} /{\text{SCF}}_{{{\text{test}}}}$				
	A	B	C	D	E	A	B	C	D	E
CS1	6.48	6.36	5.66	3.02	3.68	1.13	1.09	1.06	1.16	1.01
CS2	6.58	7.14	6.30	3.17	3.40	0.99	1.08	1.08	1.09	0.99
CS3	6.66	7.89	6.91	3.30	3.17	0.92	0.99	1.01	0.94	1.15
CS4	8.45	10.38	9.20	4.33	4.15	0.95	1.03	1.04	1.08	1.27
CS5	5.47	6.30	5.46	2.64	2.51	1.15	1.03	1.07	1.00	1.03
CS6	5.69	8.48	7.36	3.47	2.41	1.10	0.99	0.99	1.19	1.06
CS7	7.26	7.02	6.16	3.12	3.39	1.20	1.21	1.13	1.20	1.28
Average	–	–	–	–	–	1.06	1.06	1.05	1.09	1.11
Standard Deviation	–	–	–	–	–	0.101	0.070	0.045	0.090	0.112

It can be seen that the average values calculated by the proposed formulae are about 5%-11% larger than the ones tested in the experiment. Two reasons lead to this situation: firstly, the inevitable testing error, and secondly, the idealized simulation of the weld shape in Section "FE models". Nevertheless, according to the Ref.^13,16^, the proposed formulae can capture the maximum SCF in the welded tubular joints, which plays the most important role in the prediction of fatigue life and the design of the welded tubular joints. The results calculated by the proposed formulae are conservative and the formulae are acceptable for predicting the SCFs and being applied to the fatigue design of SHS-CFSHS X-joints.

### Comparison of SCF formulae between SHS-CFSHS X-joints and hollow X-joints

**Figure 35:**
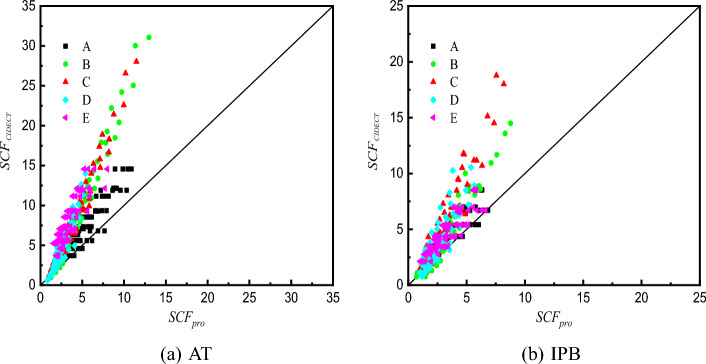
Comparison of SCFs between CIDECT formulae and proposed formulae.

For SHS X-joints, there is no concrete filled in the chord, the SCFs can be calculated by the CIDECT Design Guide No. 8^10^. In this section, the SCFs of 64 FE models in section "Parametric studies" are calculated by the formulae in Ref.^25^, and a comparison of SCFs calculated by proposed formulae in this paper and by the formulae in Ref^10^. is made, the results are listed in Table 8 and illustrated in Fig. 35.

**Table 8:** Comparison of SCFs calculated by CIDECT formula ($SCF_{{{\text{CIDECT}}}}$) and proposed formula ($SCF_{{{\text{pro}}}}$) in this paper.

	$$SCF_{{{\text{CIDECT}}}}/SCF_{{{\text{pro}}}}$$									
Specimens	AT					IPB				
	A	B	C	D	E	A	B	C	D	E
C110X8.8-B44X2.2	1.44	1.26	1.18	1.09	1.94	1.02	1.06	1.21	1.08	1.10
C110X8.8-B44X4.4	1.41	1.47	1.41	1.60	2.58	1.30	1.19	1.58	1.53	1.52
C110X8.8-B44X6.6	1.40	1.60	1.57	1.99	3.05	1.49	1.27	1.85	1.87	1.85
C110X8.8-B44X8.8	1.39	1.70	1.69	2.34	3.42	1.64	1.34	2.06	2.16	2.12
C110X6.88-B44X1.72	1.43	1.45	1.37	1.15	1.77	1.05	1.13	1.29	1.17	1.07
C110X6.88-B44X3.44	1.40	1.68	1.65	1.68	2.35	1.32	1.27	1.68	1.67	1.48
C110X6.88-B44X5.16	1.39	1.83	1.83	2.10	2.77	1.52	1.36	1.97	2.04	1.79
C110X6.88-B44X6.88	1.37	1.94	1.97	2.46	3.11	1.68	1.43	2.20	2.36	2.06
C110X5.5-B44X1.38	1.41	1.62	1.55	1.19	1.65	1.07	1.23	1.37	1.26	1.03
C110X5.5-B44X2.75	1.38	1.87	1.85	1.74	2.20	1.36	1.38	1.79	1.79	1.43
C110X5.5-B44X4.13	1.37	2.04	2.06	2.17	2.59	1.56	1.47	2.09	2.19	1.73
C110X5.5-B44X5.5	1.36	2.17	2.22	2.54	2.92	1.72	1.55	2.34	2.53	1.98
C110X4.4-B44X1.1	1.39	1.79	1.70	1.21	1.58	1.11	1.35	1.46	1.34	0.98
C110X4.4-B44X2.2	1.36	2.07	2.04	1.77	2.09	1.41	1.52	1.90	1.90	1.37
C110X4.4-B44X3.3	1.35	2.25	2.27	2.22	2.47	1.62	1.63	2.23	2.33	1.66
C110X4.4-B44X4.4	1.33	2.39	2.44	2.60	2.79	1.78	1.71	2.49	2.70	1.90
C110X8.8-B60X2.2	1.72	1.44	1.44	1.10	2.09	1.22	1.44	1.48	1.11	1.28
C110X8.8-B60X4.4	1.56	1.68	1.70	1.54	2.60	1.37	1.66	1.92	1.53	1.53
C110X8.8-B60X6.6	1.47	1.82	1.86	1.88	2.94	1.47	1.81	2.25	1.84	1.70
C110X8.8-B60X8.8	1.41	1.93	1.99	2.15	3.21	1.54	1.92	2.51	2.09	1.83
C110X6.88-B60X1.72	1.70	1.63	1.64	1.19	1.98	1.20	1.26	1.44	1.09	1.29
C110X6.88-B60X3.44	1.54	1.89	1.92	1.66	2.45	1.35	1.45	1.87	1.49	1.54
C110X6.88-B60X5.16	1.45	2.06	2.11	2.03	2.78	1.44	1.58	2.19	1.80	1.71
C110X6.88-B60X6.88	1.39	2.19	2.26	2.33	3.04	1.51	1.68	2.44	2.05	1.84
C110X5.5-B60X1.38	1.67	1.80	1.78	1.26	1.90	1.20	1.21	1.44	1.07	1.28
C110X5.5-B60X2.75	1.51	2.09	2.10	1.76	2.35	1.34	1.40	1.88	1.46	1.53
C110X5.5-B60X4.13	1.43	2.27	2.31	2.15	2.66	1.44	1.53	2.19	1.76	1.70
C110X5.5-B60X5.5	1.37	2.41	2.46	2.47	2.91	1.51	1.62	2.44	2.01	1.83
C110X4.4-B60X1.1	1.63	1.97	1.89	1.32	1.82	1.21	1.22	1.46	1.05	1.26
C110X4.4-B60X2.2	1.48	2.28	2.22	1.85	2.26	1.36	1.41	1.91	1.43	1.50
C110X4.4-B60X3.3	1.39	2.49	2.44	2.25	2.56	1.45	1.54	2.23	1.72	1.67
C110X4.4-B60X4.4	1.34	2.64	2.61	2.58	2.80	1.53	1.64	2.48	1.96	1.80
C110X8.8-B77X2.2	1.73	1.22	1.33	0.86	2.11	1.51	0.82	0.93	0.81	1.47
C110X8.8-B77X4.4	1.45	1.42	1.52	1.17	2.44	1.50	0.98	1.21	1.07	1.51
C110X8.8-B77X6.6	1.30	1.54	1.65	1.38	2.65	1.49	1.08	1.41	1.26	1.53
C110X8.8-B77X8.8	1.21	1.64	1.76	1.55	2.82	1.49	1.16	1.57	1.41	1.55
C110X6.88-B77X1.72	1.71	1.47	1.59	1.00	2.05	1.40	0.92	1.06	0.85	1.48
C110X6.88-B77X3.44	1.43	1.69	1.83	1.34	2.38	1.39	1.10	1.37	1.12	1.52
C110X6.88-B77X5.16	1.29	1.85	1.99	1.58	2.59	1.39	1.22	1.60	1.32	1.55
C110X6.88-B77X6.88	1.20	1.96	2.11	1.78	2.75	1.38	1.31	1.78	1.48	1.57
C110X5.5-B77X1.38	1.69	1.70	1.80	1.12	2.02	1.35	1.04	1.18	0.87	1.48
C110X5.5-B77X2.75	1.41	1.97	2.07	1.50	2.33	1.34	1.23	1.53	1.16	1.52
C110X5.5-B77X4.13	1.27	2.15	2.25	1.77	2.54	1.34	1.36	1.78	1.36	1.54
C110X5.5-B77X5.5	1.18	2.28	2.38	2.00	2.70	1.34	1.47	1.99	1.53	1.56
C110X4.4-B77X1.1	1.65	1.96	1.92	1.24	1.98	1.36	1.17	1.30	0.90	1.46
C110X4.4-B77X2.2	1.38	2.26	2.21	1.66	2.29	1.36	1.39	1.69	1.19	1.50
C110X4.4-B77X3.3	1.24	2.46	2.40	1.96	2.49	1.35	1.54	1.97	1.40	1.52
C110X4.4-B77X4.4	1.15	2.61	2.54	2.21	2.65	1.35	1.65	2.20	1.57	1.54
C110X8.8-B93X2.2	1.53	0.76	1.17	0.71	1.73	1.38	0.53	0.75	0.64	1.41
C110X8.8-B93X4.4	1.19	0.88	1.32	0.91	1.86	1.21	0.64	0.97	0.82	1.24
C110X8.8-B93X6.6	1.02	0.95	1.42	1.05	1.95	1.12	0.72	1.13	0.95	1.16
C110X8.8-B93X8.8	0.92	1.01	1.49	1.16	2.01	1.07	0.79	1.26	1.05	1.10
C110X6.88-B93X1.72	1.53	1.00	1.52	0.87	1.70	1.21	0.75	0.93	0.77	1.39
C110X6.88-B93X3.44	1.19	1.16	1.72	1.11	1.83	1.06	0.92	1.21	0.98	1.22
C110X6.88-B93X5.16	1.02	1.26	1.84	1.28	1.91	0.99	1.03	1.40	1.13	1.14
C110X6.88-B93X6.88	0.92	1.34	1.94	1.42	1.97	0.94	1.12	1.56	1.26	1.08
C110X5.5-B93X1.38	1.51	1.27	1.77	1.04	1.66	1.15	1.02	1.13	0.89	1.35
C110X5.5-B93X2.75	1.17	1.48	2.00	1.32	1.80	1.02	1.24	1.46	1.14	1.19
C110X5.5-B93X4.13	1.01	1.61	2.16	1.53	1.88	0.94	1.40	1.70	1.31	1.11
C110X5.5-B93X5.5	0.91	1.71	2.27	1.69	1.93	0.89	1.52	1.89	1.45	1.05
C110X4.4-B93X1.1	1.48	1.60	1.81	1.23	1.64	1.26	1.37	1.36	1.01	1.30
C110X4.4-B93X2.2	1.15	1.85	2.05	1.56	1.77	1.11	1.67	1.76	1.30	1.15
C110X4.4-B93X3.3	0.99	2.02	2.20	1.80	1.85	1.03	1.88	2.04	1.50	1.07
C110X4.4-B93X4.4	0.89	2.15	2.32	2.00	1.91	0.98	2.05	2.27	1.66	1.01
Average	1.36	1.78	1.90	1.63	2.31	1.32	1.32	1.70	1.45	1.46
Standard Deviation	0.207	0.432	0.349	0.485	0.457	0.204	0.311	0.445	0.464	0.272

It can be seen that the average values of $SCF_{{{\text{CIDECT}}}}$/$SCF_{{{\text{pro}}}}$ ratio are 1.36, 1.78, 1.90, 1.63 and 2.31 from line A to E, respectively. SHS-CFSHS X-joints have smaller maximum SCFs than corresponding hollow X-joints. This indicates that the filled concrete can effectively reduce the stress concentration by limiting the deformation of the steel tube. The maximum value is 2.31 (line E) and the minimum value is 1.36(line A). For SHS X-joints, the formulae of line A and line E in Ref^10^. are the same, and the values of $SCF_{{{\text{CIDECT}}}} { }$ are the same as well; in addition, compared with the values of line A and E in SHS-CFSHS X-joints, it can be found that line E is most affected and line A is least affected by the filled concrete, the mean values of $SCF_{{{\text{CIDECT}}}}$/$SCF_{{{\text{pro}}}}$ ratio are 2.31 and 1.36 for line E and line A, respectively. .

This result also indicates that the SCFs of SHS-CFSHS X-joint are highly overestimated if applying the formulae in the CIDECT Design Guide No.8^10^, and it is unsuitable for predicting the SCFs of SHS-CFSHS X-joint by using the method in Ref.^10^, which ignores the effect of concrete.

### Suggestions

It can be seen that the standard deviations of the AT condition are relatively small in Table 6, and in the IPB condition, the standard deviation of points A and E is also not large. In contrast, the standard deviation of points B, C, and D demonstrate significant variability, notably with point B displaying the largest standard deviation at 0.264. In Ref.^23^, some of the standard deviations of the FEA values versus the calculated values by the fitting formula are also greater than 0.2. In Ref.^16^, the maximum standard deviations in T-joint between formulae and experimental results under in-plane bending are greater than 0.2. Therefore, it can be seen that the fitting of the stress concentration coefficient has a certain degree of discreteness in the data.

**Figure 36:**
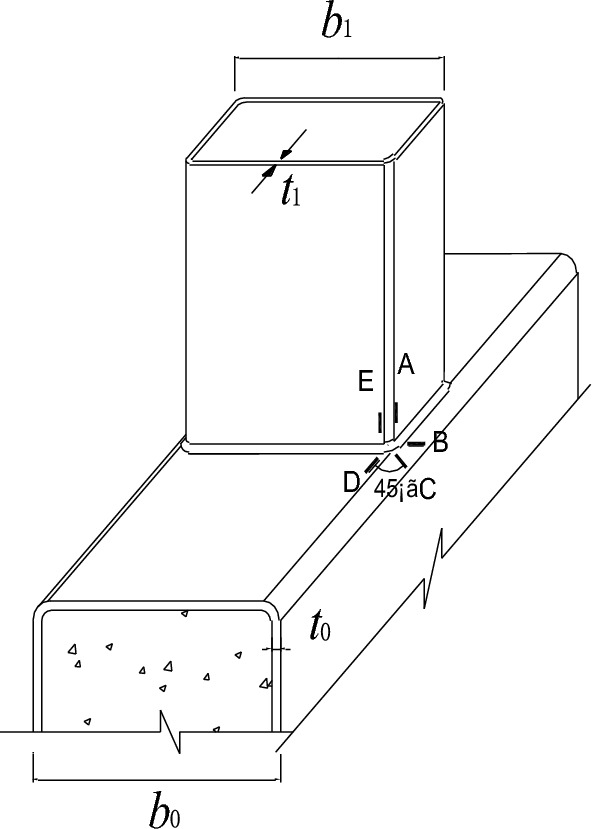
The graph of joint as β = 0.85.

Under IPB conditions, most of the data in Table 6 greater than 1.2 or less than 0.8 at points B, C, and D occur when β is equal to 0.7 and 0.85, when β is less than 0.7, the data deviations are minimal, indicating satisfactory fitting. This discrepancy arises due to increased proximity between the brace and chord edges at higher β values, leading to complex geometric and stress alterations at the weld toe positions of points B, C, and D. These changes significantly impact stress concentration factor analysis and calculation, as shown in Fig. 36. So according to this condition, the applicability of the proposed formulae (9)–(11) for predicting the SCFs of point B, C, and D in SHS-CFSHS X-joints under in-plane bending condition is limited. It is recommended to use the formulae when β does not exceed 0.7, as deviations become significant when β surpasses this threshold. Moreover, cold-formed angles and weld sizes need to be considered to determine the SCFs.

In summary, the applicability of the proposed formulae (3)–(12) for predicting the SCFs in SHS-CFSHS X-joints under axial tension and in-plane bending condition is constrained within the following ranges:

Formulae (3)–(8) and (12):

0.4 ≤ β ≤ 0.85.

12.5 ≤ 2γ ≤ 25.0,

0.25 ≤ τ ≤ 1.00.

Formulae (9)–(11):

0.4 ≤ β < 0.7

12.5 ≤ 2γ ≤ 25.0,

0.25 ≤ τ ≤ 1.00.

## Conclusion

In this paper, eight specimens were designed to assess the SCFs of SHS-CFSHS X-joints, and 64 FE models were built to make a further study on the effect of the parameters (i.e. β, γ, and τ). Based on the results of FE analysis, the SCF formulae of SHS-CFSHS X-joints were obtained by using multiple regression analysis. According to the experimental results and FE analysis in this paper, the following conclusions can be drawn:

1. The distribution patterns of hot spot stress in SHS-CFSHS X-joints under brace axial tension and in-plane bending loads were determined. For AT load condition, the maximum SCF generally occurred at line B in the chord and line A in the brace; for IPB load condition, the values of SCFs at line A and line E are closely comparable in the brace, while the maximum SCF in the chord was generally observed at line B or line C.
2. The presence of concrete within the chord enhanced its stiffness, significantly reducing maximum SCF values in SHS-CFSHS X-joints. However, it did not alter the locations of maximum SCFs. Compared to SHS X-joints without concrete, SHS-CFSHS X-joints exhibited a remarkable 33% reduction in maximum SCF during testing.
3. Parametric studies using FE analysis reveal that, for SHS-CFSHS X-joints, changes in the three parameters (β, τ, and 2γ) result in SCF variations. These phenomena are similar to those of corresponding hollow SHS X-joints as per CIDECT^25^. Notably, SCF values in SHS-CFSHS X-joints remained consistently lower than those in their hollow SHS counterparts.
4. Comparative analysis of SCFs determined through experimental tests, proposed formulae, and FE simulations indicated that the proposed formulae in this study exhibited a conservative but safer tendency. The proposed formulae consistently produce SCF values approximately 5%-11% higher than those observed in experiments. Nevertheless, these formulae remain acceptable for predicting SCFs and can be confidently applied in the fatigue design of SHS-CFSHS X-joints.
5. Importantly, this research underscored that the formulae from CIDECT^25^ overestimate the SCFs of SHS-CFSHS X-joints, rendering them unsuitable for practical prediction due to their failure to account for the concrete's effect.

## Data Availability

All data generated or analysed during this study are included in this published article.
